# Exploring Trends and Clusters in Human Posture Recognition Research: An Analysis Using CiteSpace

**DOI:** 10.3390/s25030632

**Published:** 2025-01-22

**Authors:** Lichuan Yan, You Du

**Affiliations:** College of Furnishings and Industrial Design, Nanjing Forestry University, Nanjing 210037, China

**Keywords:** human posture recognition, human activity recognition (HAR), inertial measurement unit (IMU), knowledge graph, CiteSpace, visual analysis

## Abstract

This study delves into interdisciplinary research directions in human posture recognition, covering vision-based and non-vision-based methods. Visually analyzing 3066 core research papers published from 2011 to 2024 with CiteSpace software reveals knowledge structures, research topics, key documents, trends, and institutional contributions. In-depth citation analysis identified 1200 articles and five significant research clusters. Findings show that in recent years, deep learning and sensor-based methods have dominated, significantly improving recognition accuracy, like the deep learning-based posture recognition method achieving 99.7% verification set accuracy with a 20-ms delay in a controlled environment. Logarithmic growth analysis of annual publications, supported by logistic model fitting, indicates the field’s maturation since 2011, with a shift from early simple applications of traditional and deep learning algorithms to integrating interdisciplinary approaches for problem-solving as the field matures and a predicted decline in future breakthroughs. By integrating indicators like citation bursts, degree centrality, and sigma, the research identifies interdisciplinary trends and key innovation directions, showing a transition from traditional to deep learning and multi-sensor data fusion methods. The integration of biomechanics principles with engineering technologies highlights new research paths. Overall, this study offers a systematic overview to identify gaps, trends, and innovation directions, facilitating future research and providing a roadmap for innovation in human posture recognition.

## 1. Introduction

Human posture recognition has become an essential technology with applications spanning human–computer interaction [1], intelligent monitoring [2], kinematic analysis [3], medical rehabilitation [4,5] and other fields. The goal of posture recognition is to detect and identify the positions and angles of various human body parts and joints from images or video, allowing the inference of human posture and motion. Depending on the sensors used, human posture recognition can be categorized into two main approaches: sensor-based methods and vision-based methods. This study aims to provide a comprehensive overview of major research developments from 2011 to 2024 and assess different techniques for various applications.

Sensor-based methods involve the use of wearable or externally placed sensors to collect motion data, which are then analyzed using signal processing and pattern recognition techniques to interpret body postures and movements. Common sensors, such as pressure sensors [6,7], accelerometers [8,9], gyroscopes [10], and magnetometers [10], are often combined to ensure accurate data acquisition. These methods offer the advantage of real-time 3D motion capture, independent of environmental conditions, making them suitable for complex indoor or outdoor environments. However, challenges arise from the number, placement, calibration, and synchronization of sensors, as well as the potential discomfort and interference caused to the user. Pressure sensors are particularly effective for recognizing body postures in scenarios where visual data are obstructed, such as monitoring bedridden patients under blankets. In such cases, pressure sensor arrays (PSAs) provide valuable supplementary information by measuring the pressure distribution between the body and the bed, overcoming the challenges posed by the limitations of visual systems.

Vision-based methods, on the other hand, utilize cameras or imaging devices to capture 2D or 3D visual data, which are then processed using computer vision and machine learning algorithms. These methods are non-intrusive, requiring no sensors to be worn, and are well-suited for natural or controlled environments. Nevertheless, they are sensitive to conditions like image quality, lighting, background complexity, occlusion, and self-similarity, which can complicate image analysis. Depth cameras, such as the Kinect, provide RGB-D images (which combine color and depth information), providing richer 3D data compared to monocular or stereo cameras, thereby enhancing the representation of human shape and structure.

Despite significant advances, the field still lacks unified frameworks for systematically comparing sensor and algorithm performance [11,12]. This lack of a unified framework makes it challenging to select the most appropriate methods for specific applications. A unified framework is needed to assess the accuracy, real-time performance, and scalability of various approaches, thereby guiding future research and development. In this article, we aim to review key studies from the past decade, focusing on their applications in different domains and comparing their effectiveness in various contexts. We aim to provide valuable insights and references for the continued development of human posture recognition technology.

This study conducts a comprehensive and in-depth analysis of the research landscape in human pose recognition over the past decade (2011–2024). The main research contents are as follows. Firstly, this study systematically compares and evaluates the performance of different sensor-based and vision-based methods. By understanding the strengths and weaknesses of each method, more informed decisions can be made regarding their application in specific scenarios. Secondly, this study attempts to identify the major trends and developments in the field. This includes tracking the evolution of algorithms, the emergence of new technologies, and shifting research focus over time. Finally, this study aims to uncover potential research gaps and innovative directions. By highlighting areas that require further research, it can inspire future research efforts and contribute to the continuous development of the field.

## 2. Methods

In this study, we utilized a combination of bibliometric software, statistical tools, and database search techniques to ensure accurate and comprehensive data collection and analysis. The following tools and software were employed:

### 2.1. CiteSpace as the Main Extraction and Screening Tool

CiteSpace is a widely recognized bibliometric analysis tool designed to visualize and analyze trends and patterns in the scientific literature. It was used to identify co-citation networks, research clusters, and knowledge structures in the domain of human posture recognition [13]. We input the dataset of selected articles (collected from Web of Science) into CiteSpace to analyze the network of citations between articles. The software’s built-in algorithms, such as Pathfinder Network Scaling, were applied to prune unnecessary links in the citation network, enhancing cluster clarity by removing irrelevant edges, which reduces visual clutter in citation maps.

#### Validation Through Multiple Metrics in CiteSpace

While CiteSpace’s co-citation analysis provides a powerful framework for identifying knowledge clusters, it has notable limitations, particularly in overlooking emerging but low-citation articles and interdisciplinary studies. We bridge these gaps by integrating multiple metrics below:

Citation Counts:

Citation counts are widely recognized as a fundamental measure of an article’s impact. Articles with high citation counts are indicative of their influence in shaping the research landscape and are often referenced as foundational or pivotal works within a domain.

Bursts:

In the realm of bibliometric analysis, “bursts” refer to sudden increases in the frequency of citations or occurrences of specific terms within a defined timeframe. These bursts often indicate emerging trends, shifts in research focus, or the rise of novel methodologies within a particular field. Our analysis of the dataset revealed several significant bursts that warrant detailed exploration.

Degree:

CiteSpace degree is an index used to indicate the importance or centrality of nodes in the network. It reflects the number of connections between a node and other nodes, that is, the number of its neighbors. The higher the degree, the greater the influence of the node in the network, and the more likely it is to become the source of knowledge dissemination or innovation.

Centrality:

Centrality is the intermediary centrality of a node, that is, the number of times it is on the shortest path between two other nodes. The more times a node acts as an intermediary, the higher its mediation centrality and the more important it is in the network. Centrality reflects the controlling force and bridging effect of a node. The difference between degree and centrality is that degree only considers the direct neighbors of a node, while centrality considers the indirect neighbors of a node. Both degree and centrality can be used to identify key nodes in the network, but their emphasis is different. Degree is more suitable for analyzing the local characteristics of nodes, while centrality is more suitable for analyzing the global characteristics of nodes.

Sigma (σ):

The CiteSpace sigma value is an index used to measure the novelty of nodes, which combines the importance of nodes in the network structure (intermediary centrality) and the variability of nodes in time (suddenness). The higher the centrality and suddenness of the intermediary, the higher the sigma value, indicating that the node is innovative and influential in the research field. According to the screening, the sigma metric combines centrality and citation bursts, emphasizing both the novelty and influence of an article. Articles with high sigma values are often innovative and pivotal, suggesting their significant role in advancing the research field.

By integrating these metrics, this study ensures a balanced consideration of both well-established and emerging contributions. This approach addresses potential biases or omissions inherent in relying on a single metric or method, such as co-citation analysis alone. Articles that might otherwise be overlooked due to their lower citation counts or niche focus were supplemented using these additional metrics, ensuring a more robust and inclusive dataset.

Unlike traditional studies that rely solely on citation counts or single network analysis metrics, this research innovatively integrates these multi-dimensional indicators to ensure that important literature is not overlooked. It not only focuses on highly cited literature but also captures emerging trends through the bursts indicator, measures the different roles of nodes in the network using degree and centrality, and synthesizes novelty and influence with sigma, thereby systematically outlining the knowledge structure in the field of human pose recognition. This comprehensive multi-indicator analysis method provides a more thorough and accurate research foundation for subsequent studies. Additionally, this paper compares and analyzes the latest research content with the conclusions drawn from the multi-dimensional indicators using CiteSpace, identifying the shortcomings from this perspective. At the same time, we utilize CiteSpace’s co-citation analysis to determine the main research clusters in this field, analyzing the number of research focuses in human pose recognition and uncovering the research status of each focus.

### 2.2. PRISMA as a Major Direction of Study Checklist and Guidance

PRISMA (Preferred Reporting Items for Systematic Reviews and Meta-Analyses) is a methodological framework that ensures transparency and replicability in systematic reviews. It was used to filter the large body of literature down to a manageable dataset by applying strict inclusion and exclusion criteria [14]. We used this method to guide the overall article screening.

## 3. Data Collection and Results

### 3.1. Screening Criteria

**Figure 1:**
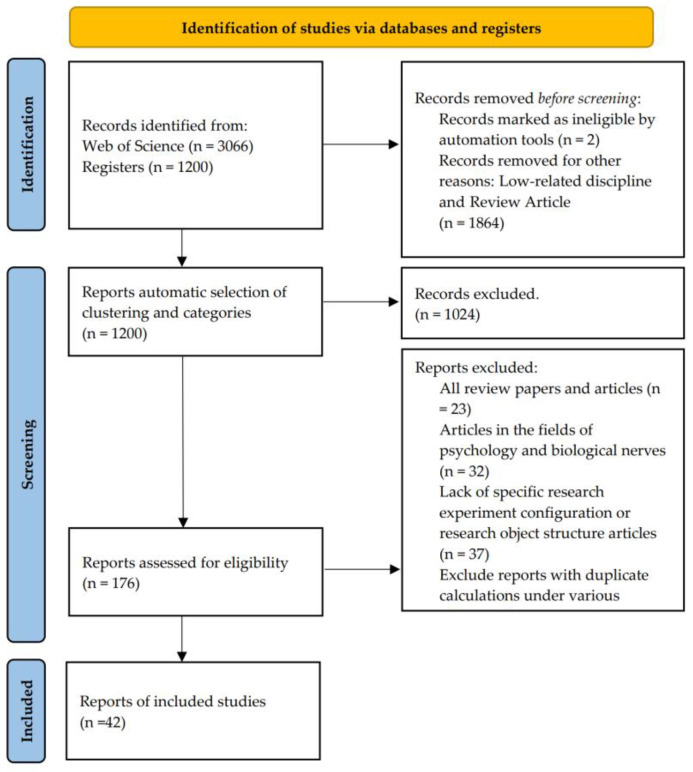
Human posture recognition literature screening process from Web of Science (2011–2024) with inclusion and exclusion criteria details.

The whole process of document retrieval is shown in Figure 1.

The Web of Science Core Collection was chosen as the article database because it comprehensively covers high-impact journals and has robust citation analysis tools, ensuring the inclusion of authoritative and highly cited articles in the field. In our preliminary article search, we found that directly using keywords such as “Human posture recognition OR Human motion capture OR Human action recognition OR Human Activity Recognition” would include a large number of psychology-related articles, which are far removed from the content of this study. Since their research area focuses on the relationship between human facial expressions and emotions and shares similarities with the search keywords used in this study, they inevitably become part of the original dataset. Therefore, we employed Boolean logic (“AND”, “OR”, and “NOT”) with the keyword combination TS = (Human posture recognition OR Human motion capture OR Human action recognition OR Human Activity Recognition) AND NOT Research Areas: Psychology OR Neurosciences Neurology, to minimize the contamination of the dataset by irrelevant fields.

The selection of the time from 2011 to 2024 aims to capture the evolution of human pose recognition technology, transitioning from traditional machine learning methods to the application of deep learning and multi-sensor fusion techniques. This timeline aligns with the emergence of key innovations in the field, such as the introduction of convolutional neural networks for pose estimation in 2014 [15].

After applying the initial search filter on the Web of Science, a total of 3066 articles were retrieved. These articles cover various aspects of human pose recognition, motion capture, and related fields. However, due to the broadness of the search terms, many articles were either irrelevant or beyond the scope of this study. Therefore, using the screening tools of the Web of Science, we automatically exclude review articles or those from unrelated fields (such as psychology and neuroscience).

In addition to using automated tools and software for data collection and analysis, a critical step in the research involved manual screening to ensure that only the most relevant articles were included in the final dataset. This process helped refine the dataset by excluding irrelevant or low-quality studies that automated tools may not adequately filter. Below is a detailed breakdown of how the manual screening was conducted:

- Relevance to Human Posture Recognition: The study must focus on human posture recognition, motion capture, or activity recognition using either sensor-based or vision-based methods.
- Technological Focus: Articles focusing on hardware (sensors, IMUs, etc.) or software (deep learning, machine learning) innovations in posture detection were prioritized.
- Exclusion of Survey or Review Papers: Papers that were purely literature reviews or meta-analyses without original research data were excluded.
- Exclusion of Articles Lacking Experimental Data: Studies that did not present specific experimental setups or lacked detailed data collection methods were removed from the dataset.
- Research Novelty: Priority was given to papers that presented innovative methodologies or introduced new technologies in human posture recognition.

### 3.2. Statistics on the Total Number of Articles Collected

**Figure 2:**
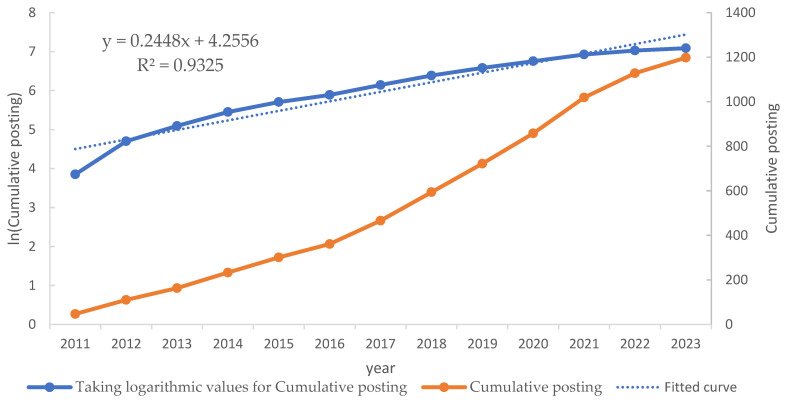
Annual publication statistics and logarithmic growth linearity analysis of 1200 selected human posture recognition articles.

A key method employed here is taking the logarithm of the annual publication count, which simplifies the visualization of growth patterns over time. When the logarithm of the publication count results in an approximately straight line, it indicates that the field follows a linear growth trend on a logarithmic scale, implying consistent exponential growth. Figure 2 shows the specific situation after taking the logarithm.

It is clear that the R-value of the logarithmic curve taken from Figure 2 with the straight line is already higher than 0.9, which indicates a high degree of fit and also implies that the raw origination data show a logarithmic growth over time. This makes it suitable for analyzing not only the changes in the number of articles sent using logarithms but also the life cycle of the field using a logistic model [16].

#### 3.2.1. Logarithmic Analysis

Linear trends in a logarithmic scale suggest exponential growth, a pattern commonly observed in scientific disciplines where publication output often accelerates before stabilizing [17,18]. Such trends are indicative of a field entering a mature stage where the rate of new findings slows down as core concepts solidify, and the focus shifts towards refinement and specialization [19].

#### 3.2.2. Logistic Model Analysis

In addition to analyzing the logarithmic growth, we applied an S-shaped logistic model (Sigmoid function) to further understand the progression of annual publication counts in human posture recognition. The Sigmoid function, often used to describe the lifecycle of research fields, provides a more nuanced view of growth trends by accounting for three distinct phases: emergence, rapid growth, and maturity. The S-shaped curve is a widely used model to describe the life cycle of scientific fields, depicting four phases: slow initial growth, rapid exponential rise, deceleration, and eventual saturation as the field matures.

The logistic growth function is mathematically expressed as:

(1) $$F\left(t\right)=\frac{k}{1+ake^{-tb}},$$

where

- $F\left(t\right)$ is the cumulative number of publications at time;
- k represents the upper limit or the maximum number of publications that the field can support (as growth slows down);
- a and b are constants that define the curve’s shape and the rate of growth; and
- $\left(t\right)$ is time (in years).

This function was fitted to the observed publication data from 2011 to 2024 using nonlinear least squares regression to estimate the parameters k, a, and b. The curve-fitting process minimizes the sum of squared residuals (SSR) to ensure the best fit for the observed data:

(2) $$SSR=\sum_{i=1}^{n}{\left(y_{i}-F\left(t_{i}\right)\right)}^{2},$$

where

- $y_{i}$ is the actual number of publications in year $t_{i}$; and
- $F\left(t_{i}\right)$ is the predicted number of publications for the same year from the fitted logistic model.

The calculated prediction curve is represented by the following equation:

(3) $$F\left(t\right)=\frac{1553}{\left(1+0.016667\times -1553\right)e^{x^{-0.348699}}}.$$

**Figure 3:**
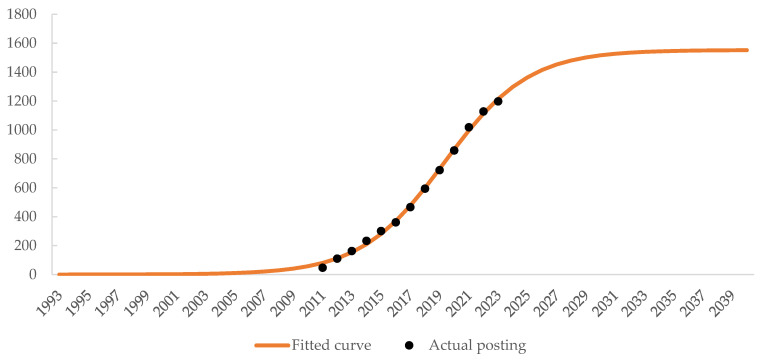
Annual cumulative and predicted publication numbers of human posture recognition documents with logistic model fitting.

Figure 3 puts the cumulative published quantity predicted by Equation (3) and the actual published quantity in the same chart.

The coefficient of determination $R^{2}$ was used to evaluate the accuracy of the fit. In this case, the model yielded a high $R^{2}$ value of 0.998, indicating that the S-shaped curve closely fits the data and is appropriate for modeling publication growth.

In our analysis, the logistic model illustrates this dynamic well. The publication trend from 2011 to 2024 shows an initial rapid rise followed by deceleration, signaling that the field of human posture recognition is approaching a mature stage. The fitted S-curve’s saturation point (defined by k=1553) suggests that the field may soon reach its upper limit in terms of significant new contributions. This indicates that major breakthroughs are likely becoming less frequent, and incremental research is taking precedence over revolutionary developments.

#### 3.2.3. The Distribution of Disciplines in the Research Literature on Human Pose Recognition

**Figure 4:**
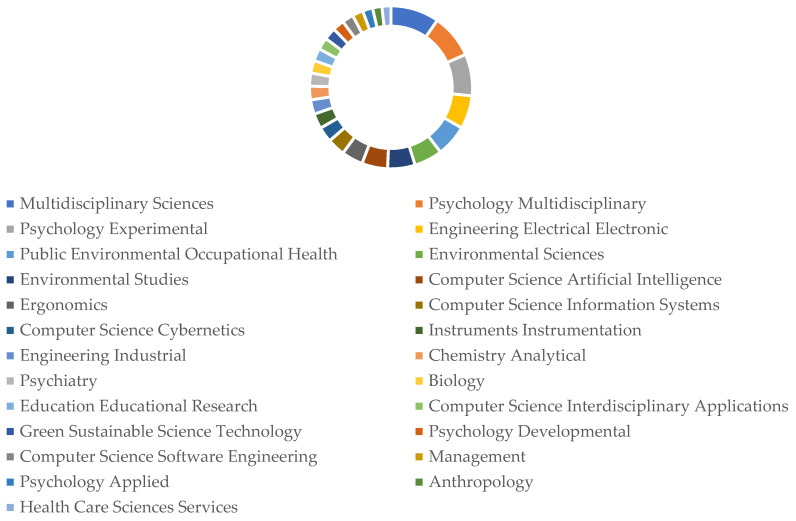
Proportion distribution of publishing fields in human posture recognition research.

As shown in Figure 4, the ring chart demonstrates that publications are not concentrated in a single subfield but span multiple disciplines. This wide distribution indicates that human posture recognition research benefits from contributions across diverse areas, reflecting the increasing collaboration between different scientific communities. Such interdisciplinary collaboration is a hallmark of a maturing research field, where advancements rely on the integration of methodologies and findings from various specialties.

### 3.3. Overlay Maps Analysis

The dual-map overlay feature in CiteSpace provides a powerful visualization of interdisciplinary research trends by mapping the relationships between citing and cited journals. This tool displays two main components: on the left, the cited journals, which represent the foundational knowledge in the field, and on the right, the citing journals, which indicate current research trends. In the picture on the left, the more papers published in journals, the longer the longitudinal axis of the ellipse; the more authors there are, the longer the horizontal axis of the ellipse is. This picture will show the theoretical basis of different topics and the current dispersion of research topics in this field as a whole [20]. By visualizing citation paths across disciplines, this method allows us to trace the intellectual flow between different scientific fields.

**Figure 5:**
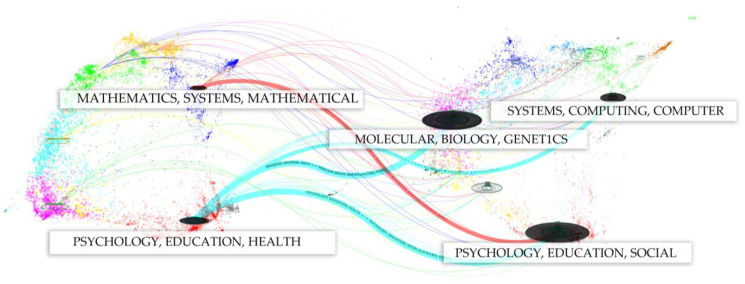
Interdisciplinary citation journal dual-map overlay in human posture recognition field.

In the field of human posture recognition, see Figure 5 for details. The dual-map overlay reveals several key interdisciplinary connections. For example, research published in journals related to engineering and computer science frequently cites foundational work in mathematics, systems science, and machine learning. This is represented by thick red and purple citation paths, indicating robust cross-disciplinary engagement between these domains.

**Figure 6:**
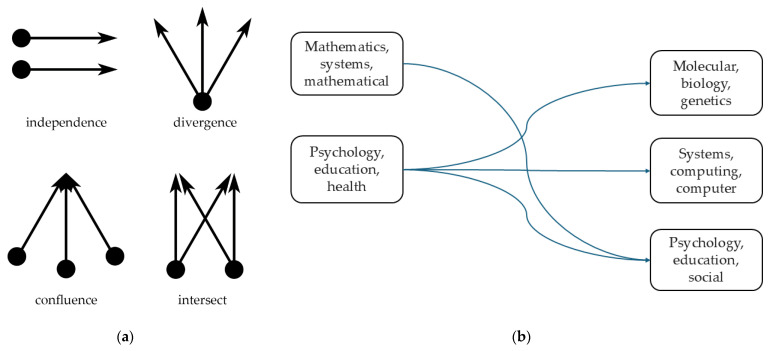
(**a**) Four citation relationships in journals proposed by Professor Chaomei Chen in human posture recognition research; and (**b**) interrelationship analysis between four main citation links and five core nodes.

Different from the extensive coverage of papers, the concentration areas of citation journals and cited journals are obviously centralized, especially in psychology and biology, where computers and mathematics appear at the same time, and there is a clear aorta to study the flow of citation data. Figure 6 refines the main reference path of double graph superposition. The main citation paths in mathematics and other research fields come from journals in psychological education and other fields alone (only the main citation paths are mentioned here). 

**Figure 7:**
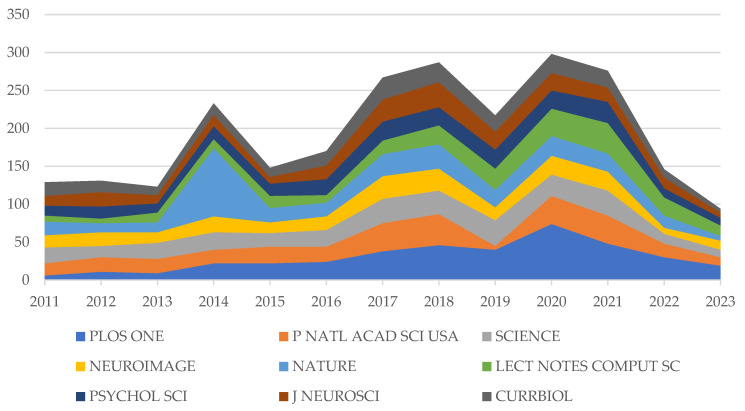
Annual citation trends of the top 10 journals in human posture recognition research.

In contrast, fields such as psychology are widely derived from three categories. This situation is more like the research that usually gathers journals in these fields. Statistics from the top 10 cited journals prove this point. Figure 7 shows that these journals with high citation volume basically show the same trend with the change of year, and there is an obvious phenomenon of common advancement and retreat, just like the three cores of psychological citation paths come together. Among them, 2014 was an abnormal point when NATURE saw a huge increase, except for PLoS One, the most cited journal. Despite the overall downward trend in 2019, the journal still maintained its level of the previous year, achieving annual increases since the statistical year. However, it still has not been able to escape the year-on-year decline that has continued since 2020. Based on the statistics on the total number of publications published in the book, research in this field has become more mature, and the decline in citations is also reflected in the above-mentioned top 10 cited journals.

### 3.4. Analysis of Co-Authorship Between Institutions

Table 1 shows that the University of California System leads with a publication count of 41, demonstrating its prominent role in advancing research. The centrality score of 0.27 signifies a prominent role within the collaborative network, highlighting that the institution is not only prolific in its publications but also acts as a pivotal hub in research collaborations. Conversely, institutions with lower publication counts, such as the Max Planck Society and the University of Toronto, exhibit lower centrality scores. This indicates that while they contribute valuable research, their collaborative impact is more limited compared to leading institutions.

**Figure 8:**
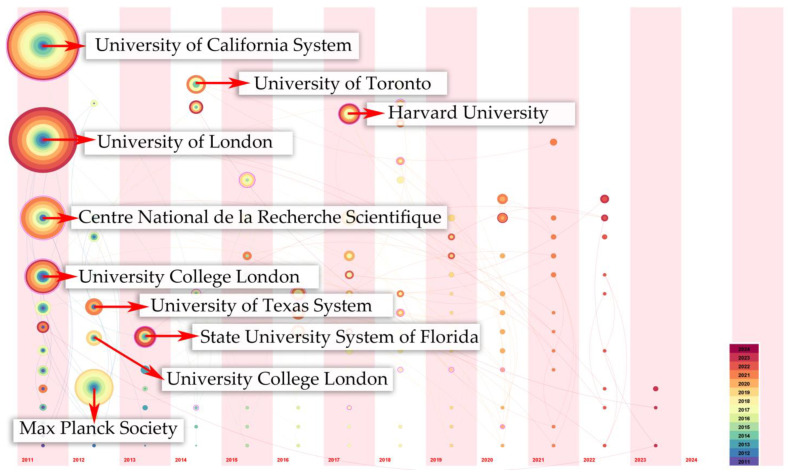
Time zone view of publication activity by research institutions in human posture recognition. Selection criteria: g-index (k = 10), LRF = 3.0, L/N = 10, LBY = 5, e = 1.0, network: N = 182, E = 161 (density = 0.0098), largest 1 CCs: 103 (56%), nodes labeled: 1.0%, Pruning: Pathfinder, Modularity Q = 0.8548, Weighted Mean Silhouette S = 0.9636.

Harmonic Mean (Q, S) = 0.9059. The time zone view in Table 1 and Figure 8 clearly shows that the earliest participating research institutions have the highest appreciation and the highest cooperation density in this field. Although Harvard University only participated in this field in 2017, it still entered the top 10 with sixth place. According to the statistics of the previous publications, 2017 is roughly in the stage of rapid development in the field of gesture recognition, which also shows that Harvard University is the main promotion institution in this field during this period, followed by Columbia University. In addition to the contribution of the two universities, the University of Arizona cannot be ignored, as it has also greatly helped the rapid development of this field.

### 3.5. Analysis of Co-Authorship Between Countries and Regions

According to Table 2, the USA leads with a total of 345 publications, followed by the UK with 153 and China with 130. The centrality metrics further highlight the USA’s dominant position in the network, indicating not only high publication volume but also influential collaborative links with other countries.

Interestingly, countries like Germany and Italy also appear as top contributors, yet their centrality scores suggest they play less of a pivotal role in the broader collaborative networks compared to the USA and UK. The centrality scores reflect each country’s role in facilitating research connections, with higher scores indicating more significant contributions to joint publications and collaborative research efforts.

**Figure 9:**
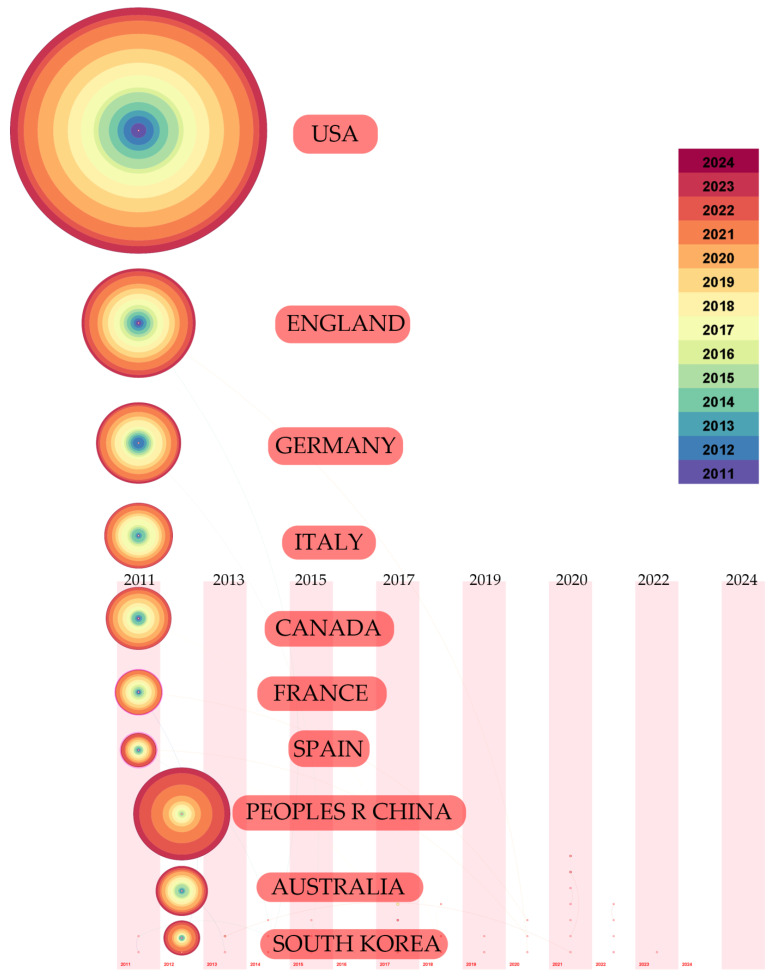
Time zone view of publication output from different countries and regions in human posture recognition research. Selection criteria: g-index (k = 10), LRF = 3.0, L/N = 10, LBY = 5, e = 1.0, network: N = 85, E = 107 (density = 0.03), largest 1 CCs: 82 (96%), nodes labeled: 1.0%, Pruning: Pathfinder, Modularity Q = 0.7536, Weighted, Mean Silhouette S = 0.8965, Harmonic Mean (Q, S) = 0.8189.

The time zone view in CiteSpace visually represents the evolution of research output from different countries and regions over time. Each node in the time zone view corresponds to a country, with the size of the node reflecting the volume of publications and its position indicating the timeline of contributions. From this view of Figure 9, it is evident that the United States stands out as the leading contributor, with consistent output since the early 2010s. Other notable contributors include China, which shows a significant increase in publication volume beginning around 2011, indicating a rapid expansion in research activity. European countries, particularly the United Kingdom and Germany, also exhibit steady growth, although their output remains comparatively lower than that of the leading countries.

This time visualization shows that the transformation of research leadership, followed by countries like China as important participants, may be affected by the increase in funds and attention to technology in academic research [21].

### 3.6. Co-Cited Literature Clustering Screening

If two or more documents are cited by one or more later documents at the same time, then these documents constitute a co-citation relationship, indicating that they have similar or related content on a certain research topic. CiteSpace can be visualized according to the co-citation relationship of documents, in which each node represents a document, and each edge represents the co-citation times between two documents. Through the visual analysis of the network diagram, we can identify the information of literature groups in different fields, the influence and emergence of literature, the connection strength between literature, and the flow of knowledge. It can help to understand the development history and present situation of a research topic, find research hotspots and frontiers, find authoritative literature and authors in the research field, and find research gaps and innovations [13]. This function was employed to screen authoritative literature across various fields.

Screening parameters:

1. Opening Pruning, Pruning sliced networks and Pruning the Merged Network. Pathfinder is to simplify the network and highlight its important structural features. The advantage of Pathfinder is completeness (unique solution), but MST (Minimum Spanning Tree) does not [22].
2. Title words are used as the cluster representation, and the figure shows the labels under the LSI algorithm.
3. The screening period is 2014–2024 (through all data deletion, it is found that the research is mainly concentrated after 2015, and the early research has little influence on the clustering formation, so the selection here is just divided into 10 slices to specifically study the results of a decade).

**Figure 10:**
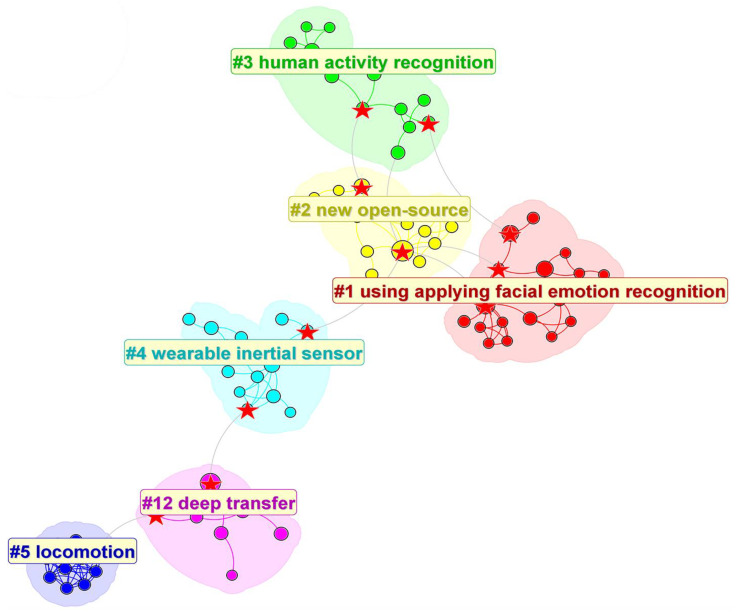
Co-citation clustering and display diagram of human posture recognition literature. Selection criteria: g-index (k = 10), LRF = 3.0, L/N = 10, LBY = 5, e = 1.0, network: N = 225, E = 303 (density = 0.012), largest 1 CCs: 75 (33%), nodes labeled: 1.0%, Pruning: Pathfinder, Modularity Q = 0.9062, Weighted Mean Silhouette S = 0.9487, Harmonic Mean (Q, S) = 0.9269. The articles with pentagram are articles that are commonly cited between two clusters.

Figure 10 illustrates the relationship between citing and cited documents. It can be clearly seen that the cited documents in these 1200 articles form six clusters. The core documents in co-cited clustering (such as cited(a) in the lower-left corner of the figure) are an important part of the knowledge base of this research field, and these documents are of great significance for understanding the research frontiers in this field. Citing(a), that is, the documents that cite the documents in co-cited clustering, are regarded as research frontiers. Citation documents usually reveal the current research trend, theme and development direction by citing the documents in co-citation clustering. These cited documents not only help to identify and understand the research trends in a specific scientific field, but also point out the potential problems and future research directions in this field [23]. According to the graph of cluster structure, it is easy to find that Clusters #12, #5, and #4 are connected with other clusters only by one article, while #1–3 are obviously more closely linked. We mark the articles that link different clusters together and will include them in the final screening article section to analyze the important contents.

**Figure 11:**
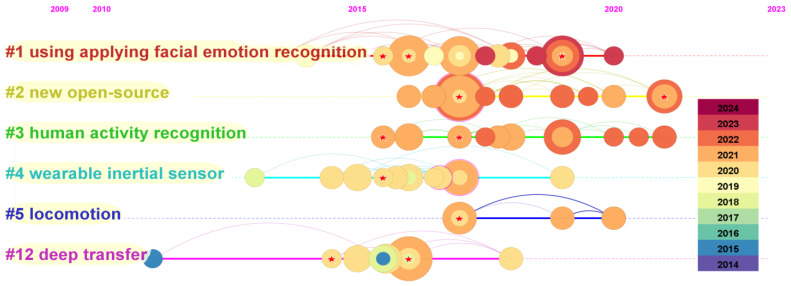
Co-citation timeline view. The articles with pentagram are articles that are commonly cited between two clusters.

The color of each annual ring in Figure 11 represents the corresponding citation time, and the thickness of the annual ring is directly proportional to the number of citations in the corresponding time division. Cluster 12 started at the earliest time but ended at the earliest time.

#### Major Clusters

According to the previous analysis, there are six important co-referenced clusters, as shown in Table 3.

Open-source Cluster 2, with 15 articles and a perfect silhouette of 1, mainly focuses on deep learning and human activity recognition, among which [24] is the most cited article. Followed by the facial emotion recognition application Cluster 1, there are also 15 articles with a silhouette of 0.973. The research mainly focuses on the emotional stage in scientific activities, and the most cited article is the research on [25]. The third cluster is human activity recognition, which also has 15 members, and the silhouette is 0.844. The focus is on activity recognition using convolutional neural networks. The main cited article is [24]. Cluster 4 is a semi-automatic working system, with 14 articles with a silhouette of 0.923. The research is related to the health and productivity of the construction industry, and the most cited article is [26]. Then there is the sustainable video surveillance system Cluster 5, with 10 articles, which also have a silhouette as high as 1. The research mainly focuses on people flow counting and tracking technology, and the main cited article is [27]. Finally, the deep migration learning Cluster 12 is introduced, with seven articles with a silhouette of 0.95, and the research focus is on the application of an accelerometer in patient monitoring, mainly citing the article [28].

According to the screening, most of the main research objects in Cluster 1 are related to human facial expressions and emotions, which are all excluded in the next screening. Each selected article has a corresponding id number (in descending order of citing or cited number). For example, in Cluster 4, the third article in the cited category is numbered id4(3)e, while the citing category is id4(3). A total of 37 articles were included across the five clusters.

Cluster 2 is clearly labeled with the same label, new open-source, under all three algorithms of CiteSpace.

Under this clustering, there is an open-source framework, OpenPose, that cannot be ignored as the first open-source real-time multi-human 2D pose estimation system. The research results on which it is based and the methods it proposes (especially Part Affinity Fields, PAFs) are highly recognized in the academic community and published in the top conference CVPR 2017, which has also extended much other research based on the framework. The framework has also extended many other studies based on it, such as the id2(2) article under the same cluster, which verified the performance of OpenPose in computing joint angles and RULA/REBA scores. It extends the use of OpenPose originally for real-time pose recognition for multiple people.

The articles in Cluster 3 focus on the use of deep learning (especially convolutional neural networks) to improve the performance of human activity recognition. They explore different network architectures and approaches that aim to improve the accuracy and efficiency of recognizing human activities through deep learning techniques. In this field, id3(1)e is the most cited one with the highest number. This article demonstrates that as the depth of deep neural networks increases, both training and testing errors may rise, not due to over-fitting but because the optimizer has difficulty in finding the optimal solution for the deep network, and the innovative nature of this article introduces the Residual Block, which significantly alleviates the difficulty of deep network optimization, making it more difficult for the optimizer to find the optimal solution of the deep network. The innovative nature of the article’s introduction of the Residual Block significantly reduces the difficulty of optimizing deep networks, allowing for better training of deeper networks, and the system based on the ResNet proposed in the article won first place in the ILSVRC and COCO 2015 competitions in several tasks, including ImageNet target detection, ImageNet localization, COCO target detection, and COCO segmentation. Although the article itself does not mention anything about human gesture recognition, it is clear that as an article in the cited category in Cluster 3, it serves to provide a knowledge base for the field of human gesture recognition. Similar to the id3(5) article, however, they compared several popular machine learning algorithms to find the most effective activity classification algorithm and ultimately chose k-NN as the optimal solution. This suggests that their work is more focused on exploring the application of traditional machine learning techniques to human activity recognition rather than relying on deep learning architectures like ResNet.

The articles in Cluster 4 basically deal with the application of Microsoft Kinect in motion tracking and analysis, or the potential use of human posture recognition in semi-automated work systems (e.g., sports rehabilitation), e.g., id4(2)e and id4(4)e explore the reliability of the Microsoft Kinect v2 in assessing standing balance and posture control and the concurrent validity. They validate the potential of Kinect V2 as a reliable and valid tool in certain static and dynamic balance tests and demonstrate that Kinect is not only suitable for gaming environments but can also provide valuable measurement data in more specialized areas such as physical therapy and rehabilitation. The other validates the effectiveness of Kinect for assessing shoulder 3D kinematics during reaching tasks in healthy adults. These articles, i.e., demonstrate that Kinect is capable of doing the job as a gaming somatosensory device with a wide range of applications in healthcare, rehabilitation training, etc. id4(2), on the other hand, uses both Kinect v2, a sensor suite containing three EXLs3 wearable IMUs, and a high-precision, multi-camera optical motion capture system in a laboratory environment (Vicon) as a reference to monitor the participant’s movement. The results show that for basic wide range-of-motion exercises in the lab setup, both systems show similarly good performance with an error range between 3 and 8 degrees. This suggests that the Kinect v2 can be used to some extent as a replacement for professional motion capture systems, especially when a low-cost solution is required. However, when it comes to more complex or subtle movements, such as those specific to certain rehabilitation exercises, the Kinect’s performance may be limited, whereas IMUs offer a higher degree of flexibility and portability, allowing for unconstrained tracking of movements (the Kinect’s solution requires the person to be directly in front of the device).

The articles that best represent the labels extracted from Clustering 5 are id5(5)e and id5(2). The authors of id5(5)e propose a new pseudo-2D stick model as well as K-ary Tree Hashing for human pose estimation and event classification. This work is particularly applicable to event recognition in daily life recordings such as sports, surveillance systems, etc. The research in this paper is directly related to “sustainable video surveillance systems” as it aims to improve the comprehension of video surveillance systems for events occurring in daily activities. id5(2) even includes the keywords sustainable video surveillance system and locomotion in the title of the article, directly pointing to the fact that this research aims to build a sustainable video surveillance system for recognizing the segmentation and movement of two people in stereoscopic action, and is an attempt to bring the technology to the ground based on what is already available.

The articles in cluster numbered 12 are centered around the deep learning approach, deep transfer learning, and the application of sensor data such as gyroscope measurement. The focus of these articles is the same as that of clustering 3 in that deep learning is used to process and analyze data from wearable devices or environmental sensors for human posture recognition and daily activity monitoring. Id12(2)e presents a framework based on deep convolution and LSTM recursive units for multimodal wearable activity recognition. This article emphasizes the importance of the ability to automatically extract features by combining convolutional layers with LSTM to capture temporal dynamic properties. This study does not rely on hand-designed features and enables natural sensor fusion, which provides a strong knowledge base for subsequent research. It addresses the problem of requiring expert knowledge to design features for traditional human pose recognition tasks and experimentally demonstrates its superior performance on different datasets. Id12(2) further explores the problem of human recognition in everyday activities, employing a DEEP transfer learning approach to address the problem of data scarcity in a multi-inhabitant environment. The authors developed a DTL-HID framework that aims to utilize limited object motion sensor data for effective individual recognition. This work not only inherits and develops previous research results on deep learning applied to human gesture recognition but also proposes innovative solutions especially for the heterogeneous data patterns specific to object sensors, which improves the model’s generalization ability and adaptability.

### 3.7. Supplementary Articles After Cluster Screening

Now merge and supplement the top 10 articles in each of the above fields with those that have been screened out before. The numbering standard is like the cluster numbering standard. For example, id3c represents the article with the third CENTRALITY in the centrality category.

In Table 4, the category “Added articles” refers to the incremental addition of articles selected from both the top 10 papers under each metric and the five major clusters. This process can result in the inclusion of articles that have already been covered under the respective metric, as these top-ranked and cluster-selected articles may overlap.

**Figure 12:**
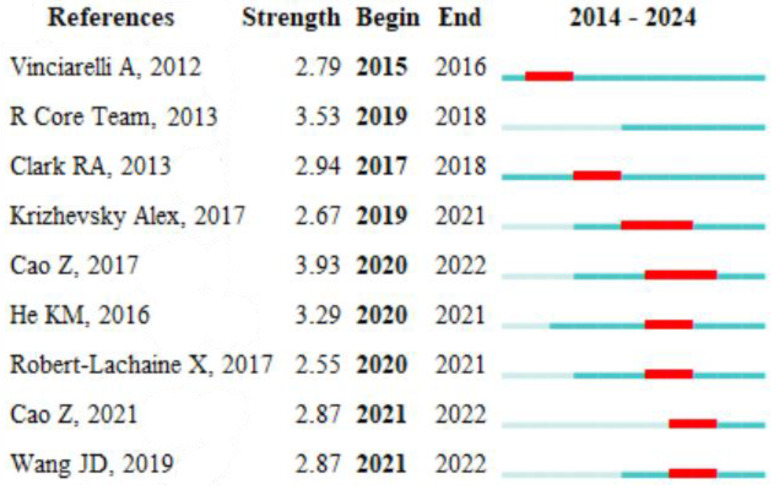
Top nine references with the strongest citation bursts in human posture recognition research and their temporal distribution [29,30,31,32,33,34,35,36,37].

Using the burst detection feature in CiteSpace, we find key terms and articles that experienced rapid citation growth. The accompanying visualization in Figure 12 illustrates the timeline of these bursts, showcasing peaks that correlate with pivotal publications in the field of human posture recognition.

The analysis revealed that certain keywords, such as “deep learning”, “human action recognition”, and “sensor fusion”, experienced notable bursts around specific years, particularly in the last five years. This aligns with the increasing integration of deep learning techniques in computer vision and posture analysis. For instance, the term “deep learning” saw a significant rise starting in 2017, coinciding with several influential papers [37,38] that introduced novel architectures for human activity recognition using convolutional neural networks (CNNs). According to the screening, the articles in this category were compared and checked, and the remaining articles with id4b were selected.

## 4. Content Analysis of the Filtered Articles

### 4.1. Trend of Selected Articles

**Figure 13:**
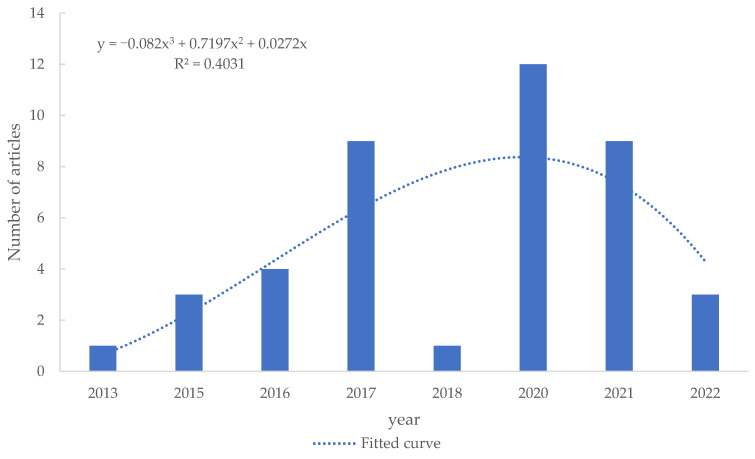
Trend analysis of selected human posture recognition articles with cubic polynomial forecast.

Obviously, from Figure 13, it can be seen that apart from the big difference between the trough in 2018 and the total number of articles collected, the trend of articles in these 10 years showed a state of first rising and then falling, reaching a peak in 2020 and then declining year by year, which still conforms to the conclusion that the development of human posture recognition field has entered a mature stage. The year 2020 is not only the peak of publishing the total collected documents but also the peak of publishing the final screened documents, which may mark a research upsurge or some important technological breakthroughs in this field.

### 4.2. Installation Position and Use Environment of Sensor

Table 5 shows the sensor locations and sampling rates used in various studies. These sensors, which are placed strategically on the body (e.g., wrists, elbows, and chest), include accelerometers, gyroscopes, pressure sensors, and RGB cameras to capture human motion effectively. For instance, study id4(6) placed Kinect v2 cameras 2 m away from the participants to track body movements, while others, such as id3(10), utilized accelerometers and gyroscopes on the chest and wrists to monitor more complex physical tasks.

A notable observation is that accelerometer and gyroscope setups dominate human activity recognition (HAR) studies, particularly when precise body joint positioning is required (Fridriksdottir et al., 2020) [28]. Studies like id4(12) confirm that using a higher number of sensors enhances accuracy, especially for joint motion tracking. The integration of wearable sensors, like pressure-sensitive insoles from the OpenGo system id2(7), provided valuable data on foot pressure during physical activities, enabling more detailed assessments of posture and gait.

#### 4.2.1. Installation Position

The selection of sensor installation position is very important in human posture recognition, and different sensors must be strategically placed based on specific application scenarios to maximize data accuracy and effectiveness. The following contents are summarized in Table 5.

Cameras: Conventional cameras and fisheye cameras are usually installed in front of participants, or the angles are adjusted according to specific needs. For example, a fisheye camera can be installed in the corner of the room to ensure that the field of vision covers the whole scene. RGB cameras may need camera calibration to ensure accurate image data at different angles.

IMU (inertial measurement unit): IMU sensors are usually installed at major joints of the human body, such as the wrist, ankle, chest, spine, arms, and legs. This installation method can accurately capture the motion data of joints and is often used for high-precision human posture analysis. For example, some systems install IMU sensors at 17 joints of the human body to capture the whole-body motion.

Smart watches: Smart watches are usually worn on the wrist and are mainly used to monitor daily activities and health indicators, such as heart rate and steps. This kind of equipment is not only convenient to wear but also portable and suitable for long-term monitoring.

GPS module: A GPS module installed in the waist or hip area is usually used to track outdoor activities and positioning and is suitable for scenes that need spatial trajectory analysis.

Pressure sensor: The pressure sensor is installed on the foot to identify gait data during walking, running and other activities. In some studies, a pressure sensor system based on insoles is used to capture the data of plantar pressure distribution, which is suitable for motion recognition and posture monitoring.

#### 4.2.2. Applicable Environment

Indoor environment:

Most sensor experiments are carried out in a strictly controlled indoor environment. Sensors commonly used for attitude recognition, such as an RGB camera, a Kinect depth sensor, and an inertial measurement unit (IMU), can reduce the interference of environmental variables in the laboratory environment, thus improving the accuracy and consistency of data. For example, the camera usually needs to be installed in a fixed position in front of the participants to ensure that it can cover the whole body or the movements of specific parts.

Outdoor environment:

In some scenes, especially when a GPS module is used for activity tracking or specific mobile tasks, sensors are deployed in outdoor environments, such as mountainous terrain or open space for monitoring. Equipment such as IMU and acceleration sensors can be used to monitor daily activities and large-scale movement without relying on visual equipment, thus overcoming the limitations of visual sensors in strong light and complex backgrounds.

Daily activity monitoring:

Some wearable devices, such as smart watches and IMU sensors, can be worn for a long time and are suitable for continuous monitoring of basic activities in daily life, such as standing, walking, running, going up and down stairs, etc. Because these devices are usually light and portable and have high real-time durability, they are suitable for environments that need long-term data acquisition.

Specific task scenario:

The use of sensors is also suitable for some specially designed activity scenes. For example, pressure sensors and IMU are often used to monitor the posture of specific tasks, such as carrying heavy objects, clumsy posture on the construction site, etc. The environment settings required for different activities usually simulate the actual scene to ensure the feasibility of the experiment and the practical significance of the data.

### 4.3. Algorithms and Identification Types

In the field of human posture recognition, various algorithms are usually used to process and analyze the collected raw data, including preprocessing, feature extraction, classification, and other operations. According to Table 6, common algorithm types can be divided into the following categories:

Deep learning (DL) algorithm: Deep learning is widely used in human posture recognition, especially the convolutional neural network (CNN), Long-term Memory Network (LSTM) and gated cyclic unit (GRU). The DL algorithm improves the recognition accuracy by learning complex patterns in data. The CNN is widely used to extract spatial features of human posture, while the LSTM is used to process time series data, which makes them perform particularly well in dynamic human posture recognition. Related research shows that the method based on the CNN is excellent in real time and accuracy, especially in the case of a large number of training data.

Traditional machine learning (CML) algorithm: Traditional machine learning algorithms such as k-NN, decision tree, and support vector machine (SVM) still have advantages in small-scale datasets and scenes with high real-time requirements. For example, SVM is often used to classify posture states and is suitable for action recognition between experts and non-experts, such as the classification id4(8)e of a bricklayer’s posture. Although CML algorithms are not as good as DL in large-scale complex scenes, they still occupy a place in some application scenarios because of their low data requirements and relatively low computational complexity.

Statistical model: The statistical model, especially the Hidden Markov Model (HMM), is superior in dealing with continuous time series and is often used for human motion recognition and posture prediction. For example, the HMM can effectively deal with the dynamic changes in human posture and has a unique advantage in identifying complex motion patterns [60].

Geometric transformation and coordinate system transformation: In 3D posture analysis, geometric transformation and coordinate system transformation algorithms play an important role. By using a quaternion rotation matrix and joint-specific Euler angle, the relative motion of different joints of the human body can be captured more accurately. This kind of algorithm is often used in bone kinematics analysis in medicine [58].

Meta-heuristic search algorithm: Meta-heuristic search algorithms, such as Binary Grey Wolf Optimization (BGWO) and Genetic Algorithm (GA), are often used to optimize the model parameters of human posture recognition. These algorithms can effectively improve the recognition accuracy in complex search space and are widely used in optimization problems [70].

**Figure 14:**
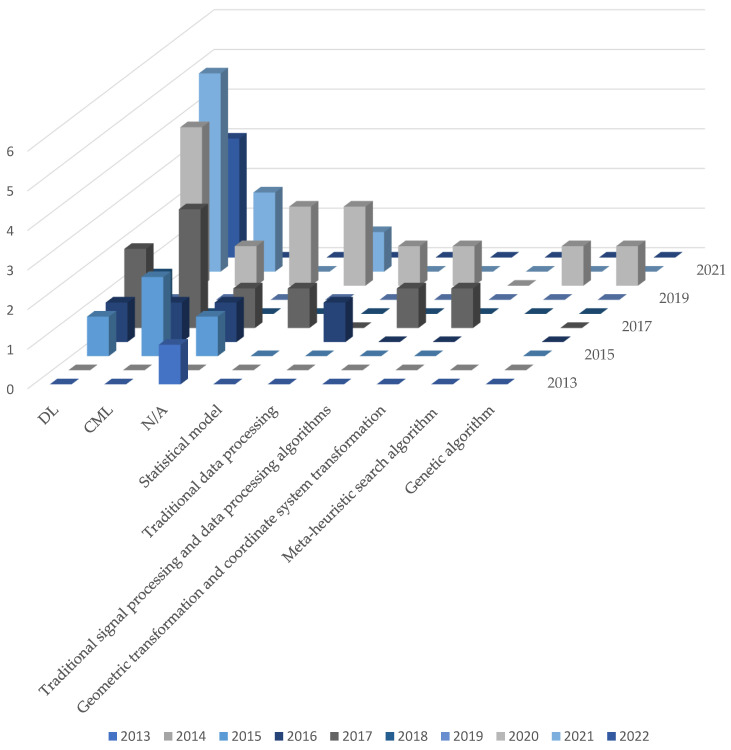
Annual distribution matrix of applied algorithms in human posture recognition research.

Figure 14 is a matrix diagram corresponding to the number and year of all the algorithms used. In the diagram, the deep learning (DL) algorithm obviously dominates the research of human posture recognition, especially reaching its peak in 2020 and 2021. This reflects the extensive application and progress of DL, including advanced models such as the convolutional neural network (CNN), Long-term Memory Network (LSTM) and GRU. This model is especially suitable for large datasets and complex recognition tasks. Although DL has made remarkable progress, traditional machine learning (CML) algorithms, such as K-nearest neighbor (k-NN) and support vector machine (SVM), still maintain strong applicability in some real-time and small datasets. In these applications, CML has a competitive advantage because of its low computational complexity and high efficiency. The frequency of other algorithms is relatively low, and they have not been used in most years, but in 2020, they will still usher in a wave of usage growth with other algorithms.

Similarly, according to Table 6, gesture recognition types are divided into two categories:

Limited content of classification: this kind of algorithm is mainly used to identify specific and limited actions or postures, such as sitting, standing, walking, running, and other basic actions. For example, the convolutional neural network (CNN) in deep learning is widely used in the classification of such limited content, and it shows efficient performance in various application scenarios.

Accurate relative position identification: This kind of algorithm aims at accurately identifying the position of various parts of the body relative to space and is suitable for more complex models and application scenarios, such as 3D skeleton modeling and posture calibration in medicine. This kind of algorithm needs to deal with more complex geometric information, and it requires higher calculation accuracy and real-time performance.

### 4.4. Computational Complexity and Accuracy of the Algorithm

Computational complexity measures the resources needed by the algorithm in the execution process, mainly including time complexity and space complexity, and describes the time and memory resources needed by the algorithm to process data, respectively. In the task of human posture recognition, especially in real-time systems, the computational complexity directly affects the efficient operation of the algorithm under specific hardware conditions. Table 7 provides statistics on the performance of filtered articles in using the dataset and the computing time complexity required to achieve the calculation results.

#### 4.4.1. Deep Learning Algorithm

The application of deep learning algorithms in human posture recognition is gradually increasing, but it also brings significant computational complexity. For example, the convolutional neural network (CNN) and Long-term Memory Network (LSTM) are excellent at processing large-scale complex data, but the calculation cost is high. The time complexity of convolution operation is usually $O\left(n^{2}\cdot d^{2}\cdot k^{2}\right),$ where n is the input dimension, d is the convolution kernel size, and k is the number of filters, depending on the number of layers of the network and the size of the convolution kernel. Take VGG-16 as an example. VGG-16 has 138 million parameters. Although it can achieve 99.7% accuracy in human posture recognition, its computational complexity and memory requirements limit its application on devices with limited resources.

Recurrent neural networks (RNNs) such as LSTM and GRU perform well in time series modeling, but due to their complex gating mechanism, the calculation complexity is high, usually $O\left(N^{2}T\right)$, where N is the number of cells in the network, T is the sequence length. In the example of id2(7), the training time of LSTM and GRU models ranges from 31 to 56 min, which shows that they have a high demand for computing resources.

#### 4.4.2. Statistical Models

Statistical models, such as the Hidden Markov Model (HMM), still have applications in traditional attitude recognition and time series processing tasks. Although its computational complexity is lower than that of deep learning algorithms, its performance is limited when dealing with complex dynamic scenes. The HMM (such as id1(4)): The time complexity of the HMM is $O\left(N^{2}T\right)$, where N is the number of states, and T is the time step. The HMM can efficiently infer the hidden state when dealing with the sequence data in human posture recognition, but its accuracy is limited when dealing with large-scale datasets. For example, in id1(4), the combination of the HMM and GOM model is applied to the attitude with limited classification, which shows relatively stable calculation performance.

#### 4.4.3. Traditional Machine Learning Algorithm

Traditional machine learning algorithms, such as k-NN and SVM, are significantly lower in computational complexity than deep learning models. The time complexity of the SVM is $O\left(n^{2}\right)$, and the complexity of k-NN is O(nk), where n is the number of samples, and k is the number of neighbors. These algorithms are suitable for small-scale datasets, but when dealing with complex high-dimensional data, their computational burden increases and their accuracy improvement is limited. For example, the SVM in id4(3)e performs well in simple classification tasks, but it is not as good as the deep learning model in the face of multi-dimensional dynamic attitude recognition.

#### 4.4.4. Geometric Transformation and Coordinate System Transformation

The importance of geometric transformation and coordinating system transformation in human posture recognition cannot be ignored, especially when dealing with three-dimensional data and posture estimation. By using a quaternion rotation matrix and joint-specific Euler angle, the algorithm can achieve accurate alignment and transformation of attitude. The computational complexity of these geometric transformations is the lowest among all algorithms, which is O(n), where n is the number of joints. When dealing with 3D data, this kind of algorithm can effectively reduce errors, especially in attitude estimation tasks. At the same time, it is often used to preprocess the raw data from sensors.

For example, in id4(11)e, geometric transformation and coordinate system transformation are used to measure the 3D joint kinematics characteristics of the human body. Although the accuracy is high, these methods depend on the accuracy and noise level of sensors when dealing with complex dynamic tasks. The geometric transformation itself does not significantly increase the complexity of the algorithm, but its performance depends on high-quality sensor data when dealing with highly dynamic scenes.

#### 4.4.5. Meta-Heuristic Search Algorithm

Meta-heuristic search algorithms (such as the Grey Wolf Optimization algorithm and genetic algorithm) are widely used in optimization problems, especially in high-dimensional search space. Compared with the traditional optimization algorithm, the meta-heuristic search algorithm can quickly converge to the suboptimal solution under uncertain conditions. Its computational complexity usually depends on the size of the search space and the number of iterations. For Genetic Algorithms (GAs), the complexity is generally $O\left(g\cdot n\cdot m\right)$, where g is algebra, n is the population size, and m is the length of each individual. The Grey Wolf Optimization algorithm (such as BGWO in id8d) also shows its potential in complex optimization problems, and its time complexity is similar to other meta-heuristic algorithms.

In the task of human posture recognition, meta-heuristic algorithms are usually used for hyperparametric optimization and feature selection of models. For example, the BGWO algorithm in id8d significantly improves the accuracy of attitude recognition by optimizing the parameters of the model. Although the computational complexity of the meta-heuristic algorithm is relatively high, its performance in a large-scale search space is better than that of the traditional optimization algorithm.

#### 4.4.6. The Relationship Between Hardware and Deep Learning

**Figure 15:**
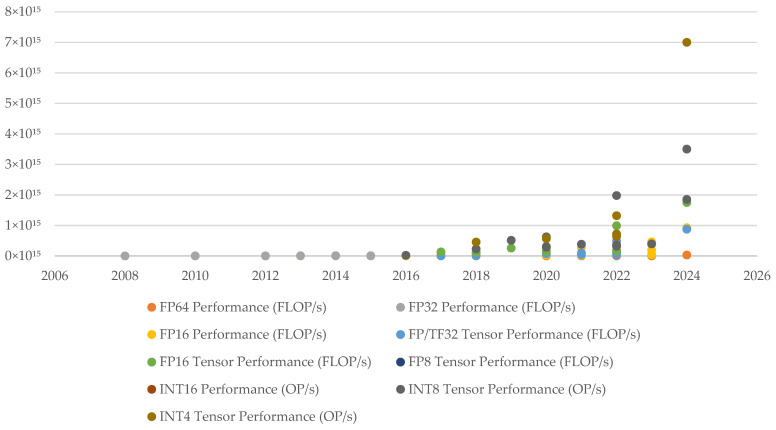
Overview of GPU computing performance development.

With the rapid improvement of GPU computing performance, the computing bottleneck of the deep learning algorithm is gradually broken. According to the results shown in Figure 15, GPU computing performance doubles every 2 to 2.5 years. In the foreseeable future, the DL algorithm accelerated by the GPU can still enjoy the technical dividend of rapid hardware iteration. In addition to pure computing performance improvement, modern GPU also supports more kinds of data formats, such as fp16, int8, and even int4, which can significantly improve performance at the expense of certain accuracy.

This progress has significantly improved the training and reasoning speed of deep learning models, enabling more complex models to be completed in a short time. With the rapid development of GPU technology, especially the significant improvement of computing power and memory bandwidth, the training time of deep learning models such as AlexNet is greatly shortened. The transition from Kepler to Volta microarchitecture reduced the training time of a single iteration by 8.9 times. In addition, by adopting advanced distributed training technology and network optimization strategies, such as GradientFlow, the training time can be significantly reduced further in the multi-GPU environment, and the training tasks that originally took days or even weeks can be completed in a few minutes. Under the scale of a 512 gpu cluster, the training time of AlexNet is compressed to an astonishing 1.5 min [75]. This not only shows the scalability of deep learning algorithms but also proves that with the continuous progress of hardware and algorithm, the same deep learning model will become easier to run.

### 4.5. Selection of Some Articles

This chapter selects and summarizes articles with constructive opinions and conclusions and discusses the limitations, trends, and directions of sensors and different algorithms in human posture recognition.

The main innovation of id2(2)e is to put forward PAFs, that is, Part Affinity Fields, which is the key technology for multi-person 2D posture estimation in this paper. The core idea of PAFs is to define a 2D vector field for each pair of body parts in the image (for example, head and neck, left hand and left elbow, etc.). These vector fields encode the spatial relationship between body parts, including their positions and directions. PAFs support the bottom-up method, which means that the model first detects all possible body parts in the image and then uses PAFs to infer the association between these parts instead of relying on the previous human detection steps. Firstly, the model detects all possible body parts (such as head, hands, feet, etc.) in the image without presupposing which specific person these parts belong to. This is the “bottom-up” part. Next, the model needs to determine how these detected parts are connected by limbs to form a complete human body. This usually involves an association strategy, such as using PAFs to represent the spatial relationship between parts. Through an analytical algorithm, such as the greedy matching or Hungarian algorithm, the model assembles the detected parts into multiple complete human postures. In this process, the model will consider the spatial consistency and correlation strength between parts. The bottom-up method does not depend on the prior human detection step. This means that the model can work directly on the image and detect and assemble the human posture without identifying all the people in the image first. Because it does not depend on human detectors, this method is more robust in occlusion, crowding, and complex human interaction scenes in images. Moreover, by centralized processing of all parts detection and correlation, repeated calculation can be reduced, and the operation efficiency of the algorithm can be improved. The model also won first place in the COCO 2016 Key Point Challenge. At the same time, OpenPose is the first open-source real-time multi-person attitude estimation system, which promotes further research and development of the research community.

The characteristic of id2(7) is that it uses a wearable insole pressure system to capture the plantar pressure data of construction workers in various working postures at the construction site and automatically extracts and classifies these data through different deep learning algorithms to identify and classify different types of awkward working postures. The OpenGo system (Motion GmbH, Munich, Germany) used in this paper includes acceleration sensors and gyroscopes, but the research focus of this paper is to use the wearable insole pressure sensor in this system to capture the plantar pressure distribution data. In the data collection stage, although the system can capture data, including acceleration, angular velocity, ground reaction force, and pressure center in the data processing stage, all data except plantar pressure mode data were removed from the dataset. It is the only one in these 42 studies that uses pure pressure data to classify human posture. This special performance brings significant advantages to specific application scenarios. Using insole pressure sensors basically interferes with the normal work of workers. In contrast, attaching multiple IMU systems to different parts of workers’ bodies will cause discomfort, and long-term work will also cause attachment position deviation. The research method can monitor workers’ working posture in real time, which provides an effective tool for preventing WMSDs and helps to improve the health and safety conditions of construction workers.

Id4(2) tested different sensor algorithms, including single sensor algorithms and sensor fusion algorithms, and compared the following specific experiments:

Accelerometer (ACC): Estimate the orientation of the sensor relative to the direction of gravity based on accelerometer data.

GYR: The dynamic attitude of the sensor is estimated by integrating the angular velocity data provided by the gyroscope.

Kalman filter (KF): Combining the data of the accelerometer and gyroscope, the Kalman filter is used to estimate the orientation of the sensor, which is an optimized sensor fusion algorithm.

Madgwick filter (MAD): This is an iterative quaternion fusion algorithm that combines the data of the accelerometer and gyroscope to provide robust attitude estimation.

Complementary filter (CF): A nonlinear complementary filter is used to combine the outputs of the accelerometer and gyroscope.

The experimental results in this paper show that the performance of sensor fusion algorithms (such as KF and MAD) is better than that of single-sensor algorithms (such as ACC and GYR). The specific performance comparison is made by calculating the error between the attitude estimation provided by each algorithm and the “gold standard” data obtained by the mark-based stereo photogrammetry motion analysis (MBS) system. The error is measured by the root mean square error (RMSE), which reflects the difference between the attitude estimated by the algorithm and the actual attitude. The results show that KF and MAD algorithms provide lower RMSE values in most cases, which means that their attitude estimation is closer to the measurement results of the MBS system. These findings show that the sensor fusion algorithm can provide more accurate attitude estimation than the single-sensor algorithm by combining information from multiple sensors. In addition, this paper also points out that although the performance of a single-sensor algorithm is slightly lower than that of a sensor fusion algorithm, in some cases, their performance decline is limited, and in some cases, they can even exceed the CF algorithm. This shows that the single-sensor algorithm can also provide acceptable accuracy under certain motion types and experimental conditions.

In addition to the single item in the IMU and the comparison of different IMUs using different algorithms, this paper also compares the advantages and disadvantages of Kinect and the IMU. Kinect has the advantage of simple operation, but its ability to track complex or high-speed dynamic motion is limited, especially when the user is not in front of the sensor. Although the IMU needs to be worn, it performs well in a variety of human movements, especially in the case of high-speed movement. The IMU can capture movement more accurately because of its high sampling rate. However, it is pointed out that Kinect can still be compared with the IMU in terms of joint angle estimation accuracy in some low dynamic movements.

The conclusion of id4(1)e shows that the RMSE of inertial sensors increases systematically in long-time and complex tasks, and the average error is 2.8 1.6, compared with 1.2 0.7 in short-term simple tasks. This shows that long-term complex actions will significantly affect the accuracy of inertial sensors (IMUs). With the complexity and duration of the task increasing, the error of the IMU also increases, which may be caused by the displacement of the sensor on the skin, the drift of the sensor, the inaccurate calibration method, and the sensitivity of the IMU to the environmental magnetic field. In addition, different environment or location changes will also introduce additional errors. Although the vision system used for reference has high accuracy, it will also be affected by soft tissue artifacts. id4(12)e also pointed out that the IMU, as a “rigid plate” fixed on the skin, may introduce different soft tissue and skin artifacts compared with MOCAP. Therefore, binding the sensor to the human body itself will bring errors.

The details of these algorithms in IMU signal processing studied by id4(12)e are unknown. Without knowing the specific working mode of the algorithms, it will be complicated to analyze the error sources and error characteristics in IMU output data. Users must rely on the technical specifications and calibration procedures provided by manufacturers, which limits the further improvement of IMU performance. In this study, despite the “black box effect”, the consistency between the IMU and MOCAP system in measuring pelvic positioning angle shows that the measurement results provided by the IMU are effective and reliable in practical applications. But, it limits the further improvement of IMU accuracy.

According to the form classification and accurate to the key points or specific postures, it can be found that it is easy to achieve high accuracy only by classifying actions, no matter what sensors and algorithms are used, but it is difficult to greatly improve the details of each state. The gap between key point detection accuracy and event classification of id5(5)e shows the gap.

According to the result of id12(2)e, convolution operation can be applied to each sensor mode interchangeably without any special preprocessing. The convolution layer can directly extract features from the original sensor data without complicated preprocessing. By fusing different types of sensor data, the model can make use of the unique information from various sensors, which is helpful to improve the identifiability of activities and reduce the misidentification caused by a single-sensor failure or inaccurate reading. The author evaluates the gesture recognition performance of DeepConvLSTM using different sensor modes on the OPPORTUNITY dataset. The F1 score of using only accelerometers is 0.689, and that of integrating accelerometer and gyroscope is 0.745 (the performance is improved by about 15%). When integrating the accelerometer, gyroscope, and magnetic sensor, it can reach 0.839, and the performance is improved by 20% on average. With an increase in the number of sensor channels, the performance of the model will continue to improve regardless of the form of the sensor. These results show that the convolution layer can directly extract features from sensor signals of different modes without preprocessing. Advantages of multimodal fusion: by fusing different types of sensor data, the model can make use of unique information from various sensors, which helps to improve the identifiability of activities and reduce the misidentification caused by a single-sensor failure or inaccurate reading.

The conclusion of id12(5)e shows that the performance of CNN in human activity identification tasks is superior to other algorithms on WISDM and UCI datasets, including basic features plus random forest (Basic features + RF), principal component analysis plus random forest (PCA + RF), and K -nearest neighbor (Segments + KNN). On the machine with the hardware configuration of Intel Xeon E5-2640 v3 8-Core CPU and NVIDIA Titan X GPU, the CNN shows a very high throughput, approaching 150,000 segments per second, which is significantly higher than other methods with fewer than 10,000 segments per second. They explain that in the prediction stage, all the operations performed by the CNN can be represented by matrix multiplication and simple threshold operation, and these operations can be parallelized very effectively on the GPU, thus making full use of the computing power of thousands of CUDA cores of the GPU. In contrast, other algorithms are mostly based on non-matrix operations, which cannot effectively accelerate the GPU in the current common implementation, so they can only rely on the computing power of the CPU. Although there are some methods to speed up the performance of these non-matrix operations, they usually require specific implementation and may not be as easy to achieve efficient parallelism on the GPU as the CNN. The author also tested the proposed solution on a moderately configured Nexus 5X Android smartphone. In this test, the CNN can process about 28 samples per second, which is enough for real-time activity recognition because the prediction is usually updated 1–5 times per second, indicating that even this smartphone released in 2015 can process in real time. In addition, it is pointed out that the ReLU activation function used by the CNN not only leads to faster learning speed because of its unsaturated characteristics but also can induce the sparsity of hidden units, thus reducing the risk of over-fitting to some extent and further improving the performance of the model.

Id5(10)e: In order to study the influence of different sensor groups and their placement positions on the accuracy of human activity recognition, an experiment was conducted. In this experiment, the sensors are placed in seven different positions of the human body, including the upper arm, elbow, wrist, chest, hip, thigh, and ankle. Through these different placement positions, the researchers explored 12 different sensor configuration combinations. Table 5 in this paper shows the comparison of the accuracy of different sensor position combinations, among which the combination of elbow, ankle, and chest shows the highest recognition accuracy on DALIAC, PAMPA2, and IM-LifeLog datasets, which are 94.23%, 94.07%, and 96.40%, respectively. The results show that the combination of sensors placed on the elbow, ankle, and chest has the best accuracy for identifying human activity classification. This discovery is of great significance for designing wearable devices and optimizing sensor layouts to improve the performance of activity recognition systems. Therefore, this paper concludes that the sensor layout of elbow, ankle, and chest positions is recommended to classify human activities more accurately. This conclusion has guiding significance for future applications in health care, physical training, safety monitoring and other related fields.

The innovation of I3D (two-stream influenced 3D Convnets) model proposed in article id1(5)e lies in its design method and the expansion of the existing architecture, including the following aspects:

Expansion design based on 2D ConvNet: The I3D model realizes the learning of temporal and spatial characteristics of video data by extending the 2D filter and pooling kernel in the depth image classification network to 3D. This design allows the model to use the pre-trained depth image classification network architecture and its parameters on ImageNet, thus achieving seamless spatio-temporal feature extraction in the video field.

Dual-stream structure (RGB and flow): The I3D model uses two independent network streams to process video data. One stream focuses on processing RGB information, and the other stream focuses on processing optical flow information (motion information of pixels in video). This two-stream structure enables the model to capture the color and motion features in the video at the same time, which complement each other and improve the performance of motion recognition. The I3D model was tested on several benchmark datasets, including UCF-101 and HMDB-51, and achieved the best performance at that time on these datasets.

The low-cost wearable IMU mentioned in id4(6) will provide unstable signals. For example, if it is set to 50 Hz, the actual data provided by the sensor will fluctuate between 20 and 90 Hz. In order to deal with this instability, a simple processing method is mentioned in this paper: deleting low-frequency window data below 40 Hz. However, this method does not cover more complex signal processing techniques, such as filtering or more advanced data interpolation methods, which shows that there is room for further research and improvement in signal processing. Moreover, this paper concludes that the Convolutional LSTM(CLN) is superior to the traditional ML-based model in recognizing workers’ postures from wearable IMU outputs because it combines CNN and LSTM structures. This is because the CLN model combines the advantages of the convolutional neural network (CNN) and Long-term and Short-term Memory Network (LSTM) and can automatically carry out feature learning and sequence pattern detection without manual feature engineering. In addition, it is also found that accelerometers contribute more to improving recognition performance than gyroscopes in gesture recognition. This shows that only accelerometer data can be considered when a trade-off needs to be made between model performance and computational efficiency. Or the data given by the accelerometer has a higher information density.

Regarding the influence of CNN layers on the model performance and calculation speed, the experimental results in this paper show that with the increase of CNN layers, the number of parameters of the model increases significantly, which may lead to over-fitting problems and increase the training and reasoning time of the model. The “shallow” CLN model mentioned in this paper (that is, CNN with fewer layers) performs well in personalized modeling, while in generalized modeling, proper CNN layers can improve the generalization ability of the model. This emphasizes the need to consider the balance between model complexity and recognition performance when choosing the DNN model architecture.

The research of id3(10) is to explore human activity recognition (HAR) in an unstructured mountain environment. In this study, a single accelerometer and deep learning model are used to develop and verify field-based data collection and evaluation method. The experimental dataset includes a variety of long-term field activities, such as running, walking, standing, lying down, climbing obstacles, etc. These activities are carried out under the conditions of changing terrain and fatigue. The experiments mentioned in this paper include the optimization of different window sizes and overlapping ratios to determine the best input parameters of the CNN (convolutional neural network) model. The window size (w) and overlap (s) jointly determine the characteristics of the data segments input into the CNN model. The window size directly affects the number of neurons used in the input layer of the model, while the overlap ratio affects the overlap degree between data segments.

In the experiment, the researcher found that the optimal window size may be different for different activity categories. For example, for some repetitive activities (such as walking and running), the choice of window size may have little effect on accuracy because these activities have a relatively fixed and periodic pattern. However, for a single activity (such as climbing obstacles), the selection of window size becomes particularly important because the data of such activities may be more variable and not repetitive.

It is mentioned in this article that when a large window size is used (for example, 1024), the recognition accuracy of most activity categories remains at a high level, but the recognition accuracy of “climb gate” is significantly reduced to 0.5, which directly affects the overall recognition accuracy. This shows that for a single activity, a smaller window size may be needed to capture its detailed features, while for repetitive activities, a larger window size can better capture its periodic features. Finally, this paper chooses a window size of 256 to obtain the highest overall recognition accuracy (0.982). This shows that under the specific conditions of this study, this is a good compromise scheme which can maintain the accuracy of repetitive activities and have sufficient recognition ability for single activities. The article does not mention other possible treatment methods, but there is indeed room for further research. For example, we can explore different machine learning models or ensemble learning methods to improve the recognition accuracy of a single activity. In addition, we can also consider using multi-sensor data, which may help to improve the ability to identify complex activities. In addition, the construction of personalized models may also be a direction because different individuals may show different characteristics when performing the same activities. This paper shows the potential of using deep learning to identify human activities in mountain environments and points out the sensitivity to window size selection under different activity categories. Future research can further explore and optimize the model on this basis in order to improve the recognition accuracy of a single activity and consider the construction of a personalized model.

Id4(8)e proposed a machine learning algorithm framework based on support vector machine (SVM), which was used to classify the working posture of bricklayers into expert and non-expert according to their working experience. The two scenarios mentioned in this paper adopt different classification strategies, respectively, and the classification accuracy and processing time of the two strategies are compared. In Scenario I, a multi-class SVM classifier is used to classify the data into four categories after preprocessing. Then the first three categories are merged into non-expert categories, and the fourth category is defined as the expert category. This method can provide more detailed classification information, such as distinguishing between novice, 1-year experience and 3-year experience workers and experts. However, this method is more complicated in calculations because it needs to calculate more class boundaries, which leads to a long processing time. As mentioned in this paper, it takes 524 s to complete the classification. Scenario II uses a binary SVM classifier to simplify the data into two categories: experts and non-experts. This method simplifies the classification process by reducing the number of categories to be classified, thus significantly reducing the processing time, which can be completed in only 13 s. Although this simplification may sacrifice some ability to distinguish different non-expert levels (such as novice, 1-year experience and 3-year experience), it has obvious advantages in computing efficiency, especially when there is a large amount of data or limited computing resources. When classifying large-scale data, simplifying the classification process can significantly improve computational efficiency. This suggests that when designing a machine learning system, we need to make a trade-off between classification accuracy and computational efficiency. In addition, it is very important to select an appropriate machine learning algorithm and adjust its parameters (such as kernel function in SVM and the number of clusters used in clustering) to optimize system performance.

Id4(9)e mentioned that their VNect and Kinect Solution occasionally make mistakes in the challenging motion prediction of crossing their legs closely, but their methods can work normally in strong illumination and sunlight, while Kinect’s depth estimation based on IR cannot work normally in this environment. VNect is a real-time 3D human posture estimation method using a single RGB camera. In contrast, VNect, as a method based on RGB information, can continue to provide effective depth estimation and attitude tracking in such an environment. Another advantage of VNect is that it can use ordinary consumer-grade RGB cameras without special depth-sensing cameras, which gives VNect potential advantages in terms of cost and popularity. The article also mentions the successful application of VNect in outdoor scenes and community videos, which are not feasible for IR-based depth cameras.

## 5. Discussion

### 5.1. Limitations of CiteSpace and Its Implications

CiteSpace primarily relies on data from the Web of Science Core Collection. This restricts the scope of the analysis to publications indexed within the Web of Science, excluding influential studies from other databases such as Scopus, PubMed, or arXiv. Moreover, it overlooks non-traditional academic contributions, such as preprints, conference papers, or software repositories, which are increasingly pivotal in fields like human posture recognition. As shown in Table 8, AlphaPose shows a significant improvement in accuracy over OpenPose in the field of human pose recognition.

**Figure 16:**
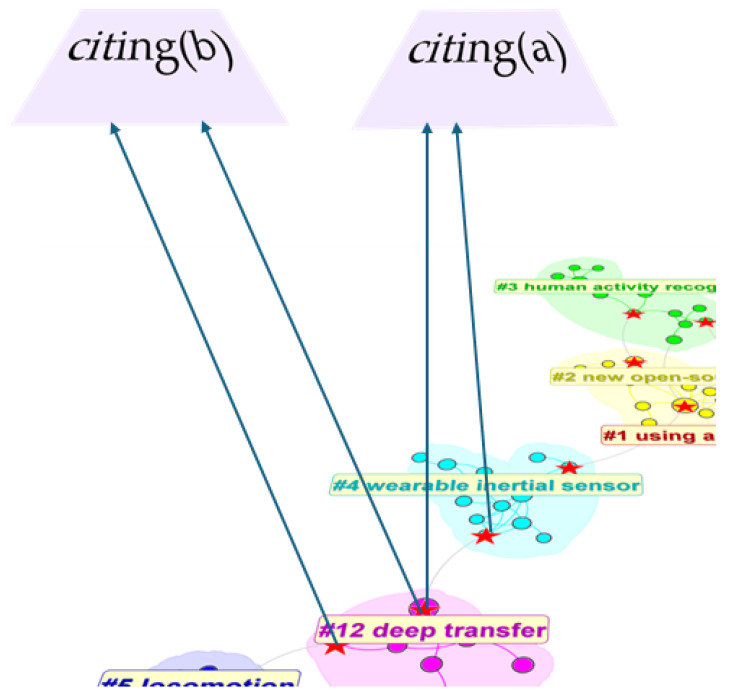
Schematic diagrams that were not included in the original dataset but were found by the screening mechanism. Where the arrow represents the reference direction. The articles with pentagram are articles that are commonly cited between two clusters.

However, such huge progress was made only 3 months apart. The OpenPose team first published version v1 on arXiv in November 2016 [35] and published at the 30th IEEE/CVF Conference on Computer Vision and Pattern Recognition (CVPR) in July 2017 at the same time as updating the article on arXiv to a v2 version for refinements, while AlphaPose’s team presented at the 16th IEEE International Conference on Computer Vision (ICCV) in October of the same year. Such a research environment motivates researchers to do their best to show their leadership in the fastest possible channel. Otherwise, their research innovations may become obsolete while waiting for the formal review process. AlphaPose, despite its widespread use and popularity in open-source communities (evidenced by Up to 8000 GitHub Stars and 2000 Forks), is not adequately represented in the literature analyzed by CiteSpace due to its reliance on formal publications rather than real-world usage metrics. Only when an article’s innovation reaches a certain level and is widely cited as the foundation of the field can it be found in CiteSpace’s co-citation link, and this paper’s OpenPose-related articles are both included in this way, as shown in Figure 16. The OpenPose articles are the cited category in the figure, and the original dataset is the citing articles above the figure. The lines connecting these two categories serve as reference paths.

### 5.2. Recent Advancements in Human Posture Recognition

Since CiteSpace relies heavily on data from the Web of Science Core Collection, this limits the ability to discover the latest research, especially those that are not yet widely cited but have a significant impact. Therefore, in this section, we focus on the latest advances within the field of human pose recognition during the last 2 years, divided into sensor technology and vision technology.

#### 5.2.1. Sensor-Based Methods

TransPose [76] is the first method to implement explicit estimation of global translations from only 6 IMUs and to compute in real time. It is the first sensor-based method to directly compute and determine the absolute position change of a human body in 3D space instead of the relative position or posture change. The framework utilizes the premise that when a person stands or moves, at least one foot (i.e., the supporting foot) remains in contact with the ground for a period and theoretically does not undergo any displacement to effectively minimize the errors due to IMU data noise and drift. This premise effectively reduces errors due to IMU data noise and drift. It means that TransPose not only focuses on the change in joint angle (i.e., posture) but further explicitly calculates the actual movement path of a person in space, which is an innovative approach realized by combining physical rules (e.g., the assumption that the supporting foot does not move) and machine learning models.

MobilePoser is likewise an influential development that enables real-time estimation of human whole-body posture and global displacement on mobile consumer devices such as cell phones, watches, and earbuds. This technology represents an advancement in a trend that has shifted from dedicated rooms and devices to arrays of wearable sensors and then, more recently, to a small number of inertial gesture capture methods. As these techniques gradually move to the use of low-precision IMUs (inertial measurement units) found on ubiquitous commodity devices such as cell phones, watches, and earbuds, a number of challenges arise, including issues of temporal consistency and loss of global displacement due to sensor noise and drift.

MobilePoser uses a multi-stage approach that combines data-driven learning with physical constraint optimization to achieve accurate motion capture. First, it utilizes a deep neural network to predict the position of each joint in the human body from IMU data. The dataset used to train this DNN is derived from synthetic IMU measurements generated by a high-quality motion capture (MoCap) system, ensuring that the model can accurately map IMU signals to specific human postures.

With this pre-training approach, the deep neural network gains optimization for human motion scenarios, which improves the understanding and parsing of complex actions. In terms of physical optimization, MobilePoser introduces human biomechanical principles, including skeletal structural constraints and laws of physics, to ensure that the estimated poses not only conform to anatomical laws but also satisfy temporal consistency and rationality of dynamic changes. These physical constraints effectively reduce the probability of misestimation under ambiguous or ambiguous motions, making the system more robust and reliable when dealing with fast or complex motions.

#### 5.2.2. Vision-Based Methods

In recent years, vision-based human posture recognition methods have made remarkable progress. Among these, YOLO (You Only Look Once) stands out as a unique model that diverges from traditional top-down and bottom-up approaches by combining object detection with key point estimation in a single-stage pose estimation solution. 

Traditionally, top-down methods first detect individual human instances and then perform keypoint estimation for each detected human (e.g., AlphaPose). On the other hand, bottom-up methods first detect all key points and then group them into individual human instances (e.g., OpenPose). Each of these approaches has its strengths and weaknesses, particularly regarding complexity and real-time performance.

YOLO Pose was implemented based on YOLOv5, and the subsequent human pose estimation features in YOLO11 adopted a different approach. They achieve both human bounding box detection and corresponding key point estimation in a single forward pass. This method eliminates the need for additional post-processing steps to group key points into individuals since each detection box is already associated with a pose. Consequently, YOLO can deliver efficient and accurate pose estimation without incurring substantial computational overhead.

YOLO11’s pose estimation can be considered a hybrid method that performs human detection and pose estimation in a single inference step. It features consistent runtime and simplifies post-processing. This design differs from typical multi-stage processing in top-down methods and from the heatmap-and-grouping strategy in bottom-up methods, aligning with YOLO’s “Only Look Once” philosophy. Such innovations make YOLO11 an efficient and versatile solution for real-time human posture recognition tasks.

Another notable advancement in this domain is HybrIK, an innovative hybrid inverse kinematics (IK) solution designed to transform accurate 3D key points into parameterized human mesh models [77]. Unlike traditional single detection models, HybrIK introduces skeletal structure constraints and IK optimization to further enhance the accuracy of 3D pose estimation.

Specifically, HybrIK uses 2D or 3D key points predicted by neural networks as input and integrates them with IK algorithms to optimize these key points, ultimately generating physically plausible human pose representations. This approach not only improves the quality of pose estimation but also enhances the realism and consistency of the results.

### 5.3. Status of Interdisciplinary Cooperation in the Field

In Figure 5 and Figure 6, we can see the broad citation paths of interdisciplinary articles, but according to the actual selected articles, few of the articles refined based on the CiteSpace filtering mechanism utilize interdisciplinary knowledge and methods to improve the accuracy and robustness of human posture recognition. Most of them are expanding the applicability of human posture recognition, such as id2(7), which selects plantar pressure as a data source for human posture recognition to reduce and prevent construction-related musculoskeletal disorders (WMSD) and accordingly cites a few articles in the field of biology. Similarly to article Id4(12)e, which focuses on the fact that posture estimation using IMUs can be applied to monitor and rehabilitate various musculoskeletal disorders, it is an assessment of the accuracy and application scenarios of the program, and in this condition, the article also cites articles from the biological field.

Still, new projects and frameworks in the last few years have utilized interdisciplinary knowledge and methods to improve human posture recognition accuracy and robustness. It would be more accurate to say that more existing information is being utilized to push the accuracy of human gesture recognition to a higher level. For example, the sensor-based solution TransPose and the vision solution HybrIK use human biomechanics. They use the human skeleton as the premise of the development of human gesture recognition in the framework of the output, which must comply with the physical constraints. MobilePoser is more advanced in the direct use of high-quality motion capture (MoCap) system-generated synthesis. MobilePoser goes one step further by directly using synthetic IMU measurements generated by a high-quality motion capture (MoCap) system for training and human skeletal structure constraints and laws of physics to further enhance the amount of raw information, which allows the framework to be used for indoor human position localization and somatosensory game play using only the information provided by the average person’s commonly used cell phone watch and wireless headset. All these projects combine their original disciplines with the biological field. Some of them explore the usability of human gesture recognition in multiple scenarios, while some of them utilize interdisciplinary knowledge to improve the accuracy of human gesture recognition itself or overcome the problem that the existing frameworks and models are not very suitable for human gesture recognition in some application scenarios.

### 5.4. Research Trends and Relevance of Industry Practices and Commercial Products

The dominance of deep learning algorithms in human posture recognition has had a profound and far-reaching impact on industry practices and commercial product development. NVIDIA’s strategic evolution in the technology landscape serves as a particularly striking illustration of this trend. With the explosive growth of deep learning applications within this field, NVIDIA, once primarily recognized for its gaming GPUs, has executed a remarkable pivot in its product design and R&D focus.

Over the past several years, NVIDIA’s financial performance has been nothing short of extraordinary. Its stock price has witnessed an astonishing surge, multiplying more than tenfold. This meteoric rise can be directly attributed to its astute positioning at the forefront of the deep learning revolution. The company’s financial reports clearly delineate a substantial and escalating commitment to research and development efforts centered around optimizing GPUs for the intricate demands of deep learning tasks. Notably, the incorporation of highly specialized tensor units within their GPUs represents a significant milestone. These tensor units have been meticulously engineered to expedite the complex mathematical operations that underpin deep learning algorithms, such as the computationally intensive CNN computations that are of paramount importance in the realm of posture recognition. Concurrently, the adoption of data formats like int8 and int4 has not only effectively curtailed memory requirements and computational overhead but has also materially enhanced the real-time processing capabilities of posture recognition systems when confronted with vast volumes of sensor data.

In the healthcare sector, the influence of deep learning has been transformative. Prominent companies such as Fitbit and Apple have seamlessly integrated state-of-the-art accelerometers and gyroscopes into their wearable devices. Leveraging the power of deep learning algorithms, these devices are now capable of meticulously analyzing body sensor data for the purpose of human activity recognition. This technological advancement has enabled a more precise identification of abnormal postures and movement patterns, which may potentially serve as early indicators of underlying health issues. In the burgeoning research area focused on the early detection of Parkinson’s disease, for instance, the analysis of minute hand tremors and body movements during routine activities has become significantly more accurate and reliable, thanks to the application of deep learning-based algorithms. These devices can continuously and unobtrusively monitor users’ walking, running, and sleep postures, thereby furnishing healthcare providers with invaluable data for proactive health management.

In the domain of intelligent manufacturing and workplace safety, the research impetus in sensor-based methods has catalyzed the development of highly practical applications. In the construction industry, industry leaders like Hilti have pioneered the development of smart workwear integrated with advanced inertial sensors and pressure sensors. Drawing directly from the research findings in this area, these wearable devices are capable of real-time monitoring of workers’ postures. They can promptly detect awkward postures and issue immediate alerts, thereby playing a crucial role in preventing work-related musculoskeletal disorders (WMSDs). Moreover, the data amassed from these sensors can be systematically analyzed to optimize work processes and training regimens, enhancing overall workplace efficiency and safety.

In the area of surveillance and security, the advancements in vision-based methods have redefined the capabilities of security cameras. Inspired by cutting-edge models like YOLO, industry giants such as Hikvision and Dahua have seamlessly integrated advanced posture recognition technologies into their security camera systems. These cameras are now empowered to conduct real-time analysis of the posture and movement patterns of individuals. They can swiftly detect suspicious behaviors such as loitering or running in restricted areas, providing law enforcement and security personnel with enhanced situational awareness and a powerful tool for maintaining public safety.

Nevertheless, the path to commercialization is not without its hurdles. The formidable computational complexity inherent in deep learning algorithms necessitates the deployment of powerful and often costly hardware. In response to this challenge, in addition to NVIDIA’s pioneering efforts, numerous other companies are vigorously exploring edge computing solutions and investing in the development of customized chips. Simultaneously, manufacturers are unremittingly dedicated to enhancing sensor reliability. In the face of environmental interference and the rigors of long-term wear, they are actively adopting advanced materials and packaging techniques and devising self-calibration algorithms to ensure the accuracy and durability of sensors.

In conclusion, the research trends in human posture recognition have unfailingly opened novel vistas of opportunity for both industry and commerce. By persistently addressing the extant challenges through collaborative innovation and technological refinement, these technologies stand poised to realize their full potential and deliver even more substantial benefits to society at large.

## 6. Results

By comprehensively utilizing CiteSpace software and various analytical indicators, an in-depth analysis of 1200 core research papers from 2011 to 2024 was conducted, thoroughly revealing the knowledge structure, research hotspots, and trends in this field, providing a systematic review and a solid foundation for subsequent research. Innovatively integrating multi-dimensional indicators such as citation counts, bursts, degree, centrality, and sigma, this study overcame the limitations of traditional single-indicator analysis, significantly reducing the probability of omitting important literature in key areas, and more accurately delineated the field’s knowledge structure, which is relatively rare in previous similar studies [13]. During the research process, the performance of sensor-based and vision-based human pose recognition methods in different application scenarios was analyzed in detail. Through the mining of a large amount of literature data, the dominant position of deep learning algorithms in this field was clarified, and the application and development trends of their integration with sensor fusion technology were deeply explored, providing valuable references for solving practical problems.

### 6.1. Summary of Characteristics of Each Sensor

Table 9 summarizes the characteristics of visual sensors, among which the RGB camera is the most widely used type of visual sensor in the field of human pose estimation. Its ubiquity is evident not only in the widespread availability of hardware devices, such as the RGB cameras embedded in smartphones and other terminal devices, but also in the transferability of its algorithms and projects. Recognition algorithms based on RGB cameras can be seamlessly adapted to analyze multimedia content, such as videos and images, on internet streaming platforms. This advantage is closely tied to the extensive use of RGB cameras in both daily life and industrial production. Over years of algorithmic iteration and development, RGB cameras have become capable of addressing the majority of human pose estimation scenarios.

In the early stages of research using RGB cameras as the information source for human pose estimation, the technology was limited to classifying a few specific human actions and was unable to localize key points of the human pose. These early methods primarily relied on manually designed features and relatively simple machine learning algorithms, which struggled to adapt to the complexity and variability of human poses, making it difficult to accurately localize key points. A significant milestone in this field can be traced back to approximately 2014, when groundbreaking progress emerged with the rise of deep learning. As deep learning evolved, research began to demonstrate the ability to estimate 2D human poses from single-frame RGB images. For instance, DeepPose and other new algorithms [78,79] marked important advancements in accurate human key point localization using RGB images.

Subsequently, in late 2016, the introduction of OpenPose represented a major milestone, providing the capability for real-time multi-person 2D pose estimation and delivering high-quality key point detection results. As for 3D pose estimation, systems such as VNect demonstrated the feasibility of real-time 3D pose estimation from monocular RGB images, with this work being published in 2017. Solutions based on single RGB cameras thus began to achieve capabilities comparable to those of depth cameras. In summary, the field of human pose estimation has evolved from merely classifying specific actions to accurately localizing human key points from RGB images, a transformation largely driven by advancements in deep learning.

In contrast, research utilizing Time-of-Flight (TOF) sensors, particularly Kinect, has also played a pivotal role in human pose estimation. Originally designed by Microsoft to break away from traditional game controllers and provide players with a hands-free gaming experience, the Kinect device was first released in 2010 for Xbox 360, followed by the v1 version for Windows in 2011 and the v2 version in 2014. These two versions served as primary sensors for research in this field. However, due to its original design purpose as a device for gaming input, Kinect faced challenges in outdoor environments with strong light, where its infrared-based depth estimation struggled to function properly.

In the early stages of exploration, such as in 2015, many studies still relied on Kinect for research. During this time, ordinary RGB camera solutions were not yet capable of obtaining or predicting depth information. The ability to directly acquire depth information through TOF sensors significantly enhanced recognition accuracy. However, as illustrated in Table 5, Table 6 and Table 7, the accuracy of RGB camera solutions steadily improved. By 2017, study id3(10) demonstrated that RGB camera solutions using the VNect algorithm had achieved performance on par with Kinect v2 while maintaining the advantage of operability in outdoor environments with strong lighting.

The multi-camera system primarily supplements the information obtained from a single RGB camera, enabling the inference of three-dimensional human positions by capturing multiple angles of the human body. Correspondingly, algorithms employing this scheme require more computational resources compared to those using a single RGB camera, as they achieve three-dimensional human pose reconstruction through camera calibration and multi-viewpoint matching. Its usage is more stringent than that of a single RGB camera, and since multiple cameras need to be placed in specific positions to capture the subject from multiple angles, it significantly restricts the subject’s range of movement. This system is typically chosen only in scenarios where high precision is required.

Similarly, we have extracted Table 10 characteristics of non-vision sensors from the articles we have filtered out. They usually have high sampling rates, easily reaching 100–200 Hz, which is a level that is difficult for ordinary vision sensors to reach, and this means that they can provide enough information during large and fast movements to make them inherently suitable for such scenarios. Moreover, accelerometers, gyroscopes, and magnetometers are usually integrated into one module. This also facilitates the installation of the sensor and the time calibration of the data. The main purpose of the scenarios using pressure sensors is to avoid affecting the normal state of the subject. For example, id2(7) is to place the pressure sensor in the insole position to roughly estimate the worker’s posture category.

When applying accelerometers to human posture recognition projects, the sensor module needs to be placed on a specific part of the body, such as a joint, which affects the normal movement of the wearer, and the need to secure the sensor to a specific part of the body by straps or other means leads to the sensor drifting away from the wearer’s initial calibrated position over a long period of time (the wearer usually has to pose in a t-shape) before the sensor can be calibrated to the initial position (the wearer usually must pose in a t-pose to calibrate the sensor’s initial position prior to use, and this calibration itself introduces errors, making it difficult to obtain a strictly accurate initial position with the involvement of the human factor), accumulating more errors as more sensors are worn over time. Moreover, due to the nature of accelerometers and gyroscopes, which require integration to obtain raw position information, projects based on the above schemes could not avoid the problem of raw data drift from the sensors, and as a result, human gesture recognition based on such schemes has only been able to obtain the relative position, but not absolute information about the human body’s key nodes in relation to space. This problem was not solved until the emergence of TransPose, which innovatively introduces the premise that when the human body stands or moves, at least one foot will remain in contact with the ground for a period of time, and theoretically, no displacement occurs, which effectively reduces the error due to the noise and drift of IMU data, and makes it possible to obtain the absolute position information. Similarly, in solving the traditional sensor installation that will affect the person’s own activities, the field also has a significant breakthrough. MobilePoser only uses a cell phone and a watch, two commonly used devices, and the installation position does not have strict requirements (watch normal wear, cell phone in the pocket, in line with the general use of habits). After a simple initial position calibration, it can be used for the input of somatosensory games like ping pong and indoor personnel location localization.

This study summarizes the characteristics of sensors, not only covering the basic performance metrics of common sensors such as RGB cameras, depth cameras, TOF sensors, pressure sensors, accelerometers, gyroscopes, and magnetometers, but also delving into their advantages and limitations under different environmental conditions and application scenarios. Particularly noteworthy is our focus on recent advancements in sensor technology, such as TransPose, which leverages the physical rules of foot-ground contact during human motion to achieve real-time estimation of global human translation from 6 IMUs, providing innovative solutions to the issues of noise and drift in inertial sensor data. Additionally, MobilePoser achieves real-time human pose and displacement estimation on mobile consumer devices by combining data-driven learning with physical constraint optimization, showcasing new breakthroughs in the application of sensor technology on portable devices. These new developments have not been sufficiently addressed or summarized in previous reviews of human pose recognition research, representing a unique contribution to this study.

### 6.2. Summary of Algorithm Characteristics

Based on the reviewed literature, this study summarizes the characteristics of different algorithms in the field of human pose estimation (as presented in Table 11).

Deep learning methods (e.g., CNNs and LSTMs) leverage complex neural network architectures with powerful feature extraction capabilities, achieving the highest levels of accuracy, especially in challenging scenarios or with large-scale datasets (e.g., multi-person pose estimation or dynamic action recognition). However, these methods are computationally intensive, particularly during the training phase, often requiring high-performance hardware such as GPUs or TPUs. Consequently, their deployment in resource-constrained environments, such as embedded systems, remains limited.

In contrast, traditional machine learning algorithms (e.g., SVMs and k-NNs) offer advantages in resource-limited and small-scale scenarios. Their moderate computational complexity and lower implementation costs make them suitable for real-time applications with lower task complexity. However, these algorithms struggle to handle high-dimensional data or complex dynamic scenarios, making them less effective for tasks requiring high precision.

Statistical models (e.g., HMMs) are primarily used for modeling time series data and dynamic action recognition. These models exhibit stable performance in capturing temporal correlations within continuous motion patterns. However, their generalizability diminishes when applied to high-dimensional or complex datasets, and they rely heavily on high-quality preprocessing and labeled data for optimal performance.

Geometric transformation methods play a critical role in preprocessing for 3D human pose estimation, particularly in mapping 2D key points to 3D space. These methods are computationally efficient and achieve high accuracy but depend on high-quality sensor data or annotations. Additionally, their applicability is limited in dynamic or diverse scenarios.

Meta-heuristic algorithms (e.g., GA and BGWO) excel in model parameter optimization and solving complex feature selection problems. These algorithms are well-suited for complex optimization tasks but are computationally expensive, especially when applied to large search spaces. As such, their utility is primarily in model development rather than real-time applications.

In summary, each algorithm type has unique strengths tailored to specific application scenarios. Deep learning methods are ideal for high-accuracy requirements in complex settings, while traditional machine learning and statistical models are better suited for small-scale or specific tasks. Geometric transformation and meta-heuristic algorithms are often employed in data preprocessing and model optimization.

## 7. Conclusions

Through a comprehensive analysis of 1200 core research papers from 2011 to 2024, it is evident that the field of human pose recognition has entered a mature stage, with deep learning algorithms becoming dominant. Whether based on visual or non-visual sensor applications, their advantages continue to expand with the annual improvement in GPU computing performance. For instance, in some complex dynamic environments, deep learning-based methods can achieve a validation set accuracy of up to 99.7% (as shown in specific research id2(1)), benefiting from their powerful learning capabilities. Meanwhile, in sensor applications, inertial sensors (such as accelerometers and gyroscopes) play a crucial role in real-time pose monitoring, and multi-sensor fusion technology demonstrates significant advantages.

However, challenges such as sensor accuracy being affected by environmental factors, difficulties in calibration and synchronization, and data synchronization persist. For example, in complex outdoor environments, environmental interferences like light changes, temperature, and humidity differences can significantly impact image recognition accuracy and sensor stability, which are critical issues that need to be addressed in practical applications. During the research process, we not only outlined the development trajectory of existing technologies but, more importantly, revealed the trend of multidisciplinary integration and potential research directions. For instance, the combination of biomechanical principles and engineering technology provides a new approach to improving pose recognition accuracy, which has been less systematically discussed in previous studies. The suggestions we proposed, such as enhancing algorithm robustness, promoting sensor miniaturization and low-power design, and establishing a unified performance evaluation framework, are based on a precise understanding of the current state and development trends of the field. These suggestions are expected to bring new breakthroughs for future research and practical applications, providing significant guidance for the further development of human pose recognition technology.

Additionally, based on in-depth analysis, this study offers forward-looking and targeted recommendations for the current state and future development trends of the field. To achieve more powerful and practical pose recognition systems, future research should focus on the following key aspects: First, efforts should be made to enhance the robustness of algorithms under different lighting and occlusion conditions, ensuring high-precision recognition in complex and changing environments through innovative algorithm design or the introduction of new technical means. For example, exploring adaptive algorithms based on deep learning that can automatically adjust model parameters according to environmental changes to improve recognition accuracy. Second, actively constructing a unified performance evaluation framework to comprehensively and objectively compare various methods and systems across different application scenarios, thereby providing scientific and effective guidance for method selection and system optimization in practical applications. This requires considering multiple factors such as accuracy, real-time performance, reliability, and cost-effectiveness to develop a set of standardized evaluation indicators and processes.

## Figures and Tables

**Table 1:** Top 10 publishing institutions.

Count	Centrality	Institutions
41	0.27	University of California System
39	0.08	University of London
25	0.36	Centre National de la Recherche Scientifique (CNRS)
22	0.02	Max Planck Society
20	0.32	University College London
12	0.1	Harvard University
12	0.14	State University System of Florida
11	0.02	University of Toronto
10	0.05	University of Texas System
9	0.04	Istituto Italiano di Tecnologia—IIT

**Table 2:** Top 10 publishing countries and regions.

Count	Centrality	Countries and Regions
345	0	USA
153	0.09	ENGLAND
130	0	PEOPLES R CHINA
114	0.05	GERMANY
92	0	ITALY
88	0.05	CANADA
70	0.05	AUSTRALIA
63	0.11	FRANCE
49	0.09	SOUTH KOREA
48	0.22	SPAIN

**Table 3:** Six important co-referenced clusters and their label under three algorithms.

Cluster ID	Size (Cited)	Size (Citing)	Silhouette	Label (LSI)	Label (LLR)	Label (MI)	Average Year
1	15	15	0.973	inquiry-based science activity	using applying facial emotion recognition (24.83, 1 × 10^−4^)	deep locality-preserving learning (0.79)	2017
2	15	8	1	new open-source	new open-source (26.48, 1 × 10^−4^)	new open-source (26.48, 1 × 10^−4^)	2018
3	15	9	0.844	human activity recognition	human activity recognition (21.13, 1 × 10^−4^)	using convolutional neural network (1.27)	2017
4	14	10	0.923	semi-automated work system	semi-automated work system (27.27, 1 × 10^−4^)	Microsoft Kinect (0.32)	2016
5	10	2	1	sustainable video surveillance system	locomotion (10.51, 0.005)	som (0.06)	2019
12	7	6	0.95	learning approach	deep transfer (22.2, 1 × 10^−4^)	gyroscope measurement (0.54)	2015

**Table 4:** Newly added articles under the five metrics with corresponding categories of inclusion.

Metrics	Remarks	Added Articles
Citation counts	High citation count signifies wide influence and recognition in the field	The top 10 articles in this category have been included in all five clusters.
Burst	Identifies key articles experiencing sudden increases in citation, indicating emerging trends or pivotal research breakthroughs	id4b
Degree	Articles with high degree values, showcasing strong connectivity and influence in the citation network	id7d, id8d, id9d
Centrality	Reflects articles acting as critical bridges in the network, connecting various research topics	The top 10 articles in this category have been included in all five clusters.
Sigma	Combines centrality and novelty, highlighting innovative and impactful studies	The top 10 articles in this category have been included in all five clusters.

**Table 5:** The comparison between the installation position of the sensor and the use environment, if a paper contains a variety of comparisons, use ① ② and other numerical serial numbers to correspond in the table.

Category	Sensor or Input	Sensor Location	Sampling Rate
id2(1) [24]	fisheye lens camera (RealSenseT265)	In a meeting corner of the room	120 Hz (120 fps)
id2(2) [39]	① 3 FLIR Blackfly S cameras ② Microsoft Kinect v2 ③ Xsens MVN Link inertial MoCap system	① Located directly in front of the participant and 40 degrees in front of the left and right (camera calibration is required) ② 2.5 m directly in front of the participant ③ Located in 17 joints of the human body	① 10 Hz ② 30 Hz ③ 240 Hz (Xsens system)
id2(3) [40]	RGB image	N/A	N/A
id2(4) [41]	BioMed bundle motion capture system (52 IMUs)	Spine, arms, legs	90 Hz
id2(7) [42]	OpenGo system (16 capacitive pressure sensors, a 3-axis gyroscope, and a 3-axis accelerometer)	Foot	50 Hz
id2(1)e [33]	RGB image	N/A	N/A
id2(2)e [32]	RGB image	N/A	N/A
id2(3)e [43]	① ActiGraph GT9X Link ② Zephyr BioHarness™3	① Head, shoulder, center-waist, Non-dominant-side waist ② Chest, under armpit	100 Hz
id2(5)e [44]	Video	N/A	N/A
id2(12)e [45]	RGB-D image	N/A	N/A
id3(3) [46]	GPS module and GY-521 module (including angular acceleration measurement, angular velocity measurement, angle measurement, and temperature)	Right hip	Wifi reporting frequency: 1 Hz
id3(5) [47]	Smartwatch FTW6024 by Fossil (Acceleration and angular velocity sensors)	Ankle	50 Hz
id3(10) [48]	BioHarness (3-axis acceleration) Garmin Forerunner GPS watch	Around the chest	BioHarness (100 Hz) Watch (1 Hz)
id3(11)e [49]	Gyroscopes and accelerometers	N/A	N/A
id4(2) [50]	① Kinect v2 ② Wearable IMU (EXLs3 by Exel srl, Italy)	① Was placed 3.5 m in front of the subject ② Thorax and of left thigh and shank	① Kinect v2 30 Hz ② 100 Hz
id4(3) [51]	Kinect v2 Three-axis gyroscope and three-axis accelerometer	Wrists and ankles	N/A
id4(4) [52]	Kinect V2	2 m away in front of the subjects	30 Hz
id4(5) [53]	Kinect V2	N/A	30 Hz
id4(6) [54]	Accelerometer and gyroscope	Multiple joint nodes	20–90 Hz (40 Hz was used as a cut-off to remove lower-frequency window)
id4(1)e [35]	① Xsens ② Eight-camera Optotrak system	Located in 17 joints of the human body	① 240 Hz ② 30 Hz
id4(2)e [55]	① Kinect V2 ② 3DMA system	① 2.5 m directly in front of the participant	① 30 Hz ② 100 Hz
id4(3)e [56]	Accelerometer and gyroscope sensors built in smartphones	Upper arm	100 Hz
id4(4)e [57]	Kinect V2	Optical axis parallel to participants’ sagittal plane (front), 15° to the left side of the participants, or 30° to the left of the participants	30 Hz (Spline interpolation upsamples data to 60 Hz)
id4(6)e [58]	① Kinect V1 ② Kinect v2	Directly in front of the subject	60 Hz
id4(7)e [59]	① Accelerometer and gyroscope ② OMC system	N/A	20 Hz ② 80 Hz (Linear interpolation down sampling to 20 Hz)
id4(8)e [60]	Xsens MVN	The position of the center of 28 body joints	100 Hz
id4(9)e [61]	RGB image	N/A	N/A
id4(11)e [62]	Gyroscopes, magnetometers, and accelerometers	Head, chest, bilateral upper arms and forearms	80 Hz
id4(12)e [63]	① Accelerometers, gyroscopes, and magnetometers ② MOCAP (6 VICONMX-3+ and 2 VIcON MX-T20 cameras and one Kistler 9281A pressure plate)	Pelvis	① 100 Hz ② 200 Hz
id5(1)e [64]	Depth camera	N/A	N/A
id5(3)e [65]	Depth Sensors	N/A	N/A
id5(5)e [66]	RGB image	N/A	N/A
id5(10)e [67]	Three IMU sensors (3-axis accelerometer, 3-axis gyroscope and 3-axis magnetometer) and an NRFL0	chest, elbow and ankle	N/A
id12(2) [68]	① OPPO: accelerometer ② HANDY: accelerometer, gyroscope, magnetometer	N/A	① 30 Hz ② 52 Hz
id12(2)e [69]	5 RS485-networked XSense(IMU) 2 InertiaCube3 inertial sensors 12 Bluetooth acceleration sensors	XSense (IMU) included in a custom-made motion jacket, 2 InertiaCube3 inertial sensors located on each foot 12 Bluetooth acceleration sensors on the limbs	30 Hz
id12(5)e [70]	Accelerometer and gyroscope	N/A	50 Hz
id12(7)e [71]	Accelerometer	Wrists, chest and ankles	100 Hz
id4b [31]	① Kinect ② 3DMA (Vicon Nexus V1.5.2 with 12 Vicon MX cam)	① Within about 3.5 m from Kinect	① Fluctuation around 30 Hz ② 120 Hz
id7d [72]	Gyroscope and accelerometer	N/A	N/A
id8d [73]	Three GY-521 accelerometer sensors	Arms, legs and neck	N/A
id9d [74]	Three-axis accelerometer, gyroscope and magnetometer	Chest, thighs and wrists	100 Hz

**Table 6:** Recognition types, algorithms, and their categories in human posture recognition research.

Category	Recognition Range	Recognition Type	Algorithm Category	Arithmetic
id2(1) [24]	Eating, reading, operating a smartphone, operating a laptop computer, and sitting	Classify limited content	DL	DL (VGG-16 + OpenPose) Optimization algorithms: Adam
id2(2) [39]	Global 3D human skeleton posture	Accurate relative position of components to space	DL	① open pose
id2(3) [40]	Throwing, Relying, Lying, Jumping, Helmet	Classify limited content	DL	DL (Faste R-CNN)
id2(4) [41]	① TV assembly ② Airplane assembly ③ Glassblowing ④ Motions based on EAWS	Classify limited content	Statistical model	GOM model HMM
id2(7) [42]	5 different types of awkward working posture	Classify limited content	DL	① LSTM ② Bi-LSTM ③ GRU
id2(1)e [33]	Accurate to every movement of the human body	Accurate relative position of components to space	DL	DL: Part Affinity Fields (PAFs)
id2(2)e [32]	Accurate to every movement of the human body	Accurate relative position of components to space	DL	DL: OpenPose
id2(3)e [43]	Trunk posture	Classify limited content	N/A	N/A
id2(5)e [44]	N/A	Classify limited content	CML	Two-Stream Inflated 3D ConvNets (I3D)
id2(12)e [45]	Crossing, talking, walking, queueing, waiting	Classify limited content	CML	Preprocessing: (geodesic distance, 3D Cartesian plane, way-point trajectory and joints MOCAP) feature optimization: Particle Swarm Optimization (PSO) classifier: Neuro-Fuzzy Classifier (NFC)
id3(3) [46]	Sit, stand, walk, run, fall, lay on one’s back, lay face down, lay on	Classify limited content	CML	CML (k-NN)
id3(5) [47]	Standby Forefoot strike Midfoot strike Rearfoot strike	Accurate relative position of components to space	DL	Feature-Based Learning: NB RF SVM dl: LSTM GRU Conv1D Optimization algorithms: Adam
id3(10) [48]	In the mountain environment Lay, sit, climb gate, walk, run	Classify limited content	DL	DL (CNN)
id3(11)e [49]	Various daily activities, including walking, running, going up and down stairs, etc.	Classify limited content	DL	DL (CNN-GRU)
id4(2) [50]	Accurate to every movement of the human body	Accurate relative position of components to space	① CML ② Traditional signal processing and data processing algorithms	① Kinect SDK ② Kalman filter (KF) Madgwick filter(MAD) Complementary filter (CF)
id4(3) [51]	Shoulder range-of-motion	Accurate relative position of components to space	N/A	N/A
id4(4) [52]	Body joint coordination patterns	Accurate relative position of components to space	N/A	N/A
id4(5) [53]	Evaluate the joint angle of the upper limb during daily functional tasks	Accurate relative position of components to space	DL	dl (LSTM)
id4(6) [54]	A variety of worker postures	Classify limited content	DL	dl (Convolutional LSTM)
id4(1)e [35]	Accurate to every movement of the human body	Accurate relative position of components to space	Traditional signal processing and data processing algorithms	① MVN model (Xsens built-in)
id4(2)e [55]	Accurate to every movement of the human body	Accurate relative position of components to space	N/A	N/A
id4(3)e [56]	Construction posture classification	Classify limited content	CML	① Neural network ② Decision tree ③ K-nearest neighbor (KNN) ④ Logical regression ⑤ Support vector machine (SVM)
id4(4)e [57]	Shoulder movement	Accurate relative position of components to space	CML	Kinect for Windows SDK 2.0
id4(6)e [58]	Joint center locations during static postures	Accurate relative position of components to space	CML	① Kinect for Windows SDK 1.5 ② Kinect for Windows SDK 2.0
id4(7)e [59]	Accuracy and repeatability of measuring torso angular displacement and upper arm elevation	Accurate relative position of components to space	Traditional data processing	Complementary weighting algorithm
id4(8)e [60]	Proficiency in brick-moving posture	Classify limited content	CML	SVM
id4(9)e [61]	Global 3D human skeleton posture	Accurate relative position of components to space	DL	Vnect (CNN)
id4(11)e [62]	3D joint kinematics	Accurate relative position of components to space	Geometric transformation and coordinate system transformation	Quaternion rotation matrix and joint-specific Euler angles
id4(12)e [63]	Measure dynamic pelvic positioning angle	Accurate relative position of components to space	N/A	N/A
id5(1)e [64]	Accurate to every movement of the human body	Accurate relative position of components to space	Statistical model	Preprocessing: floor removal object segment 3-D CCL human detection human identification HMM
id5(3)e [65]	Interactions between people, such as handshakes and hugs	Classify limited content	Statistical model	Feature extraction and optimization: codebook, GMM, fisher encoding, cross-entropy optimization function classification; MEMM
id5(5)e [66]	Accurate to every movement of the human body	Accurate relative position of components to space	Traditional data processing	Pseudo-2D stick model Ray Optimization K-ary Tree Hashing
id5(10)e [67]	Daily movements, such as lying or standing	Classify limited content	CML	Filter: Chebyshev, Elliptic and Bessel Optimizer: e Probability Based Incremental Learning (PBIL) Classifier: K-Ary Tree Hashing
id12(2) [68]	Activities of Daily Living (ADLs)	Classify limited content	DL	CNN-based DTL-HID
id12(2)e [69]	Recognition modes of locomotion and postures recognition of sporadic gestures	Classify limited content	DL	DeepConvLSTM
id12(5)e [70]	Walking Upstairs Downstairs Sitting Standing Lying	Classify limited content	DL	CNN
id12(7)e [71]	Lying down, sitting, standing, walking, running, cycling, Nordic walking, ascending stairs, descending stairs, vacuum cleaning, ironing clothes, jumping rope	Classify limited content	CML DL	① KNN ② Rotation forest ③ Neural network
id4b [31]	Gait assessment	Accurate relative position of components to space	N/A	N/A
id7d [72]	The activities of the human body in different situations	Classify limited content	Statistical model	Optimization algorithm: Adam and AdaDelta Post-processing: MEMM
id8d [73]	The activities of the human body in different situations	Classify limited content	Meta-heuristic search algorithm	Optimization algorithm: Binary Grey Wolf Optimization (BGWO) Classifier: Decision tree (DT) classifier
id9d [74]	Different modes of human activity	Classify limited content	Genetic algorithm	Genetic algorithm, GA

**Table 7:** Computational complexity and performance of algorithms in human posture recognition research.

Category	Dataset	Performance	Computational Time Complexity
id2(1) [24]	MPII Human Pose Dataset human posture dataset and the common objects in context (COCO) key points challenge data	99.7% (validation dataset)	N/A
id2(2) [39]	10 Korean young males	Xsens as reference ① The RMSE for all joint angles is 8.4° ② The RMSE for all joint angles is 13.4°	③ 20 ms delay
id2(3) [40]	Specific action pictures from 31 students	The model’s accuracy in detecting throwing, relying, lying, jumping actions, and wearing helmets was 90.14%, 89.19%, 97.18%, 97.22%, and 93.67%, respectively.	N/A
id2(4) [41]	N/A	F-Score (%) ① 96.84 ② 94.33 ③ 94.70 ④ 91.77	N/A
id2(7) [42]	10 participants	Accuracy (%) ① 97.99 ② 98.33 ③ 99.01	Training time ① 31 min ② 56 min ③ 54 min
id2(1)e [33]	① MPII human multi-person dataset ② COCO 2016 key points challenge dataset	① 75.6% mAP ② 60.5% AP The champion of that year	N/A
id2(2)e [32]	① MPII human multi-person dataset ② COCO 2016 key points challenge dataset	① 75.6% mAP ② 65.3%	Nvidia 1080 Ti and CUDA 8 ① 73 ms ② 74 ms
id2(3)e [43]	Ten samples from one subject	Mean Bias (°) ① Chest Ref Back 0.3 Waist 5.7 Head −0.5 Shoulder −3.7 Center–waist 4.6 ② Chest 1.2 Under armpit −1.2	N/A
id2(5)e [44]	Kinetics (Pre-training) ① HMDB-51 ② UCF-101	① 80.2% ② 97.9%	N/A
id2(12)e [45]	① NTU RGB + D ② UoL 3D social activity dataset ③ Collective Activity Dataset (CAD)	① 93.5% (NTU RGB + D dataset) ② 92.2% (UoL dataset) ③ 89.6% (Collective Activity Dataset)	A Core i5-4300U CPU is used to compute the running time. For one frame, the computational time for recognition of human action was 0.11 s.
id3(3) [46]	Dataset from collected nine elderly activities with 11,000 records	0.964	N/A
id3(5) [47]	Approximately 23.7 h running and walking data	NB: 72 scores RF: 83 scores SVM: 93 scores LSTM: 94 scores GRU: 94 scores Conv1D: 96 scores	NB: 130 s RF: 139 s SVM: 134 s LSTM: 47 s GRU: 46 s Conv1D: 28 s
id3(10) [48]	Contains 3,341,184 samples covering activities at various terrain and fatigue levels	0.978	May not be feasible for battery-powered field devices
id3(11)e [49]	① UCI-HAR ② WISDM ③ PAMAP2	① 96.20% ② 97.21% ③ 95.27%	N/A
id4(2) [50]	From three subjects	② Slightly better than ①; both errors in the range of 3 to 8 degrees for all the joint angles	N/A
id4(3) [51]	50 asymptomatic adults	All free and fixed AROM. This system demonstrated adequate reliability (ICC ≥ 0.7).	N/A
id4(4) [52]	45 healthy participants	The mean and standard deviation of the PoV entropy feature is 2.602.	N/A
id4(5) [53]	13 healthy male university students	shoulder and elbow flexion/extension waveforms with mean CMCs > 0.93 shoulder adduction/abduction, and internal/external rotation waveforms with mean CMCs > 0.8	N/A
id4(6) [54]	4 workers	Macro F1 score 0.870	On (Intel Core i7-7700 CPU@ 2.8 GHz, 16 GB RAM, NIVIDA GeForce GTX 1060 GPU@16 GB RAM system) recognizes 256 postures per second
id4(1)e [35]	12 healthy participants	① long complex task mean ± SD RMSE on all joints was 2.8° ± 1.6° short simple tasks was 1.2° ± 0.7° ② as reference	N/A
id4(2)e [55]	30 healthy participants	① Single leg test: ICC value range = 0.70 to 0.80 Double leg test: 0.44 to 0.47 ② as reference	N/A
id4(3)e [56]	2 participants	① 62–95% ② 63–94% ③ 68–96% ④ 65–96% ⑤ 63–94%	N/A
id4(4)e [57]	17 healthy participants	RMSE of shoulder flexion and extension is less than 10 °	N/A
id4(6)e [58]	20 participants	For upright standing posture, average error ① 76 mm ② 87 mm	N/A
id4(7)e [59]	10 dairy workers	① The RMSD ranges from 4.1 to 6.6° for the torso and from 7.2 to 12.1° for the upper arm ② as reference	N/A
id4(8)e [60]	21 participants with different professional levels	① scenario1 91.23% ② scenario2 92.04%	Processing time (seconds) ① 524 ② 13
id4(9)e [61]	① MPI-INF-3DHP ② Human 3.6 m	Mean Per Joint Position Error (MPJPE) ① 80.5 mm ② 124.7 mm	Capable of working in real time at a frequency of 30 Hz on a single TitanX (Pascal architecture) system
id4(11)e [62]	6 surgical faculty members	The neck and torso flexion/extension angles are accurate to 2.9 ± 0.9 degrees and 1.6 ± 1.1 degrees, respectively. Shoulder elevation is accurate to 6.8 ± 2.7 degrees. Elbow flexion is accurate to 8.2 ± 2.8 degrees.	N/A
id4(12)e [63]	17 healthy participants	① The range of anterior sagittal pelvic angle RMSE is 2.7 °−8.9 ° ② as reference	N/A
id5(1)e [64]	① IM-DailyDepthActivity ② MSRAction3D ③ MSRDailyActivity3D	① 72.86% ② 93.3% ③ 94.1%	N/A
id5(3)e [65]	① SBU Kinect Interaction ② UoL3D Social Activity ③ UT-Interaction	① 91.25% ② 90.4% ③ 87.4%	N/A
id5(5)e [66]	① UCF50 ② hmdb51 ③ Olympic sports dataset	Key points detection accuracy ① 80.9% ② 82.1% ③ 81.7% Event classification accuracy ① 90.48% ② 89.2% ③ 90.83%	N/A
id5(10)e [67]	① DALIAC ② PAMPA2 ③ IM-LifeLog	① 94.23% ② 94.07% ③ 96.40%	N/A
id12(2) [68]	Two real-world ADL datasets are gathered: “Opportunity” (OPPO) and HANDY	OPPO: Micro-averaged 0.707 HANDY: F-1 Score 0.984	N/A
id12(2)e [69]	① OPPORTUNITY dataset ② Skoda	F1 score ① Modes of Locomotion 0.895 Gesture Recognition 0.915 ② 0.985	N/A
id12(5)e [70]	① WISDM ② UCI-HAR	① 93.32 ② 97.63	On Nexus 5X smartphone (cpu only), the system was able to classify about 28 samples per second. On Xeon E5-2640 v3 8-Core CPU and NVIDIA Titan X GPU, 149,600 samples per second.
id12(7)e [71]	PAMAP2	① 0.890 ② 0.941 ③ 0.900	N/A
id4b [31]	21 healthy adults	② As reference Gait speed, step length: r and rc values > 0.9 Foot swing velocity: r = 0.93 rc = 0.54 stride time: R^2^ = 0.991	N/A
id7d [72]	① USC-HAD ② IMSB ③ Mhealth human dataset	① 91.25% ② 93.66% ③ 90.91%	N/A
id8d [73]	① MOTIONSENSE ② MHEALTH ③ IM-AccGyro human–machine dataset	① 88.25% ② 93.95% ③ 96.83%	N/A
id9d [74]	① IM-WSHA ② WISDM ③ IM-SB ④ SMotion	① 81.92% ② 95.37% ③ 90.17% ④ 94.58%	N/A

**Table 8:** The 2015 COCO test-dev human posture estimation results under different algorithms.

Method	AP @ 0.5:0.95	AP @ 0.5	AP @ 0.75	AP Medium	AP Large
OpenPose (CMU-Pose)	61.8	84.9	67.5	57.1	68.2
Detectron (Mask R-CNN)	67.0	88.0	73.1	62.2	75.6
AlphaPose	73.3	89.2	79.1	69.0	78.6

AP (Average Precision), e.g., AP @ 0.5:0.95 indicates that the AP value is the average precision calculated over the range of IoU (Intersection over Union) thresholds from 0.5 to 0.95 (in 0.05 steps).

**Table 9:** Summary of visual sensors properties.

	Visual Sensors		
Category	RGB Camera	Depth Camera	TOF
Subdivision category		Multi-camera system	Time-of-Flight (ToF) (mainly represented by Kinect)
Sensor characteristics	No need for structured light, TOF transmitters and receivers, relatively low hardware costs, relying on natural light, can be used indoors and outdoors	Without the need for structured light, ToF emitters, and receivers, the hardware cost is relatively low. It relies on ambient light and can be used both indoors and outdoors. Multi-camera systems, which rely on visual features for image matching, have complex matching algorithms.	The measurement range of ToF is relatively long and unaffected by surface grayscale and features, and the depth distance calculation remains stable at the centimeter level without varying with distance.
The directness and indirectness of sensor measurement	Indirect	Indirect	Unlike multi-camera systems that require algorithmic processing to output three-dimensional data, ToF can directly output the three-dimensional data of the measured object.
Sampling rate	Moderate sampling rate	The sampling rate is the lowest among all schemes, which is not suitable for high-speed movement.	Low to medium sampling rate
Applicable environment	Strong and dim light conditions have a significant impact. It captures less information compared to multi-camera systems.	Strong and dim light conditions have a significant impact.	It is essentially unusable under strong outdoor light conditions.
Continuous working hours	No need for attachment installation, and there is no deviation in the sensor attachment position due to prolonged operation.		
Layout position	Usually installed in front of the person	Need a specific angle to cover the part of the body	Usually installed in front of the person
Influence on human body movement after use	None		
Highest recognition type	Accurate relative position of components to space		

**Table 10:** Summary of non-visual sensors properties.

	Non-Visual Sensors			
Category	Pressure Sensor	Accelerometers	Gyroscopes	Magnetometers
Sensor characteristics	The architecture exhibits a straightforward design, characterized by rapid responsiveness and elevated sensitivity.	The system is simple, responsive, and sensitive, but integrating and filtering data can lead to cumulative errors.	High sensitivity, fast response, but with significant zero drift, poor stability, and the need for filtering and integration that can lead to cumulative errors.	Not affected by gravity, no need for integration.
		The three are often used in combination with each other.		
Amount of information provided	Low	In non-visual schemes, this sensor can provide the most human body posture information.		
Sampling rate	It generally has a high sampling rate, making it more suitable for high-speed motion detection.			
Applicable environment	It is susceptible to the influence of environmental factors such as temperature and humidity and requires calibration and compensation.			It is easily affected by gravity and noise.
Continuous working hours	No need for attachment installation, and there is no deviation in the sensor attachment position due to prolonged operation.	Prolonged operation can lead to displacement of the wearing position and drift of the sensor itself, which can significantly affect the accuracy of sensors, making them unsuitable for continuous long-term work.		
Influence on human body movement after use	It will have little impact on users	Installed on specific parts and joints of the body		
Highest recognition type	Classify limited content	Accurate relative position of components to space		

**Table 11:** Summary of algorithm characteristics.

Algorithm Type	Computational Complexity	Accuracy	Applicability	Advantages and Disadvantages
Deep learning (CNN, LSTM)	High	Highest	Best for large, complex datasets	High accuracy but resource-intensive, especially in devices with limited resources.
Traditional machine learning (SVM, k-NN)	Moderate	Moderate	Small-scale datasets, real-time tasks	Efficient on small datasets but less accurate on high-dimensional or large data.
Statistical models (HMM)	Moderate	Moderate	Time series processing, continuous motion recognition	Good for handling dynamic motion, but limited when facing complex datasets.
Geometric Transformation	Low	High (for 3D data)	Preprocessing in 3D posture analysis	Effective in reducing error but relies on high-quality sensor data for dynamic scenarios.
Meta-heuristic algorithms (GA, BGWO)	High	High	Complex optimization tasks, feature selection	Optimizes model parameters effectively but computationally expensive, especially in large-scale search spaces.
